# The Post-Translational Modifications, Localization, and Mode of Attachment of Non-Covalently Bound Glucanosyltransglycosylases of Yeast Cell Wall as a Key to Understanding their Functioning

**DOI:** 10.3390/ijms21218304

**Published:** 2020-11-05

**Authors:** Valentina V. Rekstina, Tatyana A. Sabirzyanova, Fanis A. Sabirzyanov, Alexei A. Adzhubei, Yaroslav V. Tkachev, Irina B. Kudryashova, Natalia E. Snalina, Anastasia A. Bykova, Alice V. Alessenko, Rustam H. Ziganshin, Sergei A. Kuznetsov, Tatyana S. Kalebina

**Affiliations:** 1 Department of Molecular Biology, Faculty of Biology, Lomonosov Moscow State University, Moscow 119991, Russia; vrextina@gmail.com (V.V.R.); plotta@inbox.ru (T.A.S.); biophoenix@mail.ru (F.A.S.); newsmol2009@yandex.ru (I.B.K.); winny9797@mail.ru (A.A.B.); 2 Engelhardt Institute of Molecular Biology, Russian Academy of Sciences, Moscow 119991, Russia; alexei.adzhubei@gmail.com (A.A.A.); yygmf@runbox.me (Y.V.T.); 3 Emanuel Institute of Biochemical Physics, Russian Academy of Sciences, Moscow 119334, Russia; volhared30@gmail.com (N.E.S.); alicealessenko@gmail.com (A.V.A.); 4 Shemyakin and Ovchinnikov Institute of Bioorganic Chemistry, Russian Academy of Sciences, Moscow 117997, Russia; rustam.ziganshin@gmail.com; 5 Institute of Biological Sciences, University of Rostock, 18059 Rostock, Germany; sergei.kuznetsov@uni-rostock.de

**Keywords:** cell wall, glucanosyltransglycosylases, post-translational modifications, microcompartments, Bgl2, Scw4

## Abstract

Glucan linked to proteins is a natural mega-glycoconjugate (mGC) playing the central role as a structural component of a yeast cell wall (CW). Regulation of functioning of non-covalently bound glucanosyltransglycosylases (ncGTGs) that have to remodel mGC to provide CW extension is poorly understood. We demonstrate that the main ncGTGs Bgl2 and Scw4 have phosphorylated and glutathionylated residues and are represented in CW as different pools of molecules having various firmness of attachment. Identified pools contain Bgl2 molecules with unmodified peptides, but differ from each other in the presence and combination of modified ones, as well as in the presence or absence of other CW proteins. Correlation of Bgl2 distribution among pools and its N-glycosylation was not found. Glutathione affects Bgl2 conformation, probably resulting in the mode of its attachment and enzymatic activity. Bgl2 from the pool of unmodified and monophosphorylated molecules demonstrates the ability to fibrillate after isolation from CW. Revealing of Bgl2 microcompartments and their mosaic arrangement summarized with the results obtained give the evidence that the functioning of ncGTGs in CW can be controlled by reversible post-translational modifications and facilitated due to their compact localization. The hypothetical scheme of distribution of Bgl2 inside CW is represented.

## 1. Introduction

A rigid cell wall (CW) is essential for protection of the yeast cells against negative environmental factors and plays a significant role in the stress response [1,2].

Complex of polysaccharides covalently linked to mannoproteins is a natural mega-glycoconjugate (mGC) that plays the central role as the CW structural component [1,2]. The main polysaccharide part of mGC consists of β-glucan (about 35–55%). The minor polysaccharide part of mGC is represented by chitin (about 1.5–6.0%), also being an important structural component [1].

There are 30–50% of mannoproteins in the CW. They are highly mannosylated molecules with a protein part of 4–5% [1]. Mannose chains are attached to asparagine (N) and serine/threonine (S/T) residues by way of N- and O-mannosylation respectively [2]. CW mannoproteins can be divided into two groups: covalently attached to glucan as a part of mGC and non-covalently attached to mGC that can be extracted from the CW by treating with SDS under heating.

The group of non-covalently attached mannoproteins includes glucan remodeling enzymes [1,2,3,4] responsible for forming of mGC–glucanosyltransglycosylases (ncGTGs). These ncGTGs are essential for the protection of yeast cells against heating, drying and impact of inhibitors of CW assembly such as Congo Red, Calcofluor White and SDS [5,6,7,8,9]. There are 4 ncGTGs in the CW: Bgl2, Scw4, Scw10 and Scw11. Among them the major ncGTGs are Bgl2 and Scw4 [5,7,8,9,10].

Bgl2 is a major, constitutive and conservative mannoprotein not only in many species of yeast, but also in other fungi [11]. Bgl2 isolated from *Saccharomyces cerevisiae* CW demonstrated amyloid-like properties [12,13]. Bgl2 amino acid sequence has two potential sites of N-glycosylation (N-x-S/T), N202 and N284, and only one of them could be N-glycosylated [14]. Still there are contradictory data on N-glycosylation in the literature. Authors of two unrelated studies found that N-glycosylation occurs on different sites [15,16].

Scw4 is also a constitutive and conservative mannoprotein among different fungi species [11]. It is interesting that minor share of Scw4 molecules was recently identified as covalently linked mannoproteins [17,18]. Scw4 has also single N-glycosylation, determined at N89 position [19].

In the absence of Bgl2 and Scw4 some GPI-anchored CW mannoproteins are not able to integrate into CW or attach correctly [8,10]. Also deletions of *BGL2* gene cause defects in CW assembly and the increase of the content of chitin in *S. cerevisiae* CW [20]. This compensatory response can be defined as “chitin repair” mechanism. *Ogataea parapolymorpha* (*Hansenula polymorpha*) cells, that have low level of chitin reparation after *BGL2* deletion, are characterized by disturbed formation of the bud scar [21]. In mutant cells this area contains cytoplasmic cell material and numerous membrane invaginations [21]. Simultaneous deletion of *SCW4* and *SCW10* or *SCW11* genes demonstrates slower growth rates and morphological abnormalities of the yeast cells [6].

There is some data showing that various non-covalently bound proteins, ncGTGs in particular, can be extracted to varying degrees by various extractants used. At least part of Bgl2 can be extracted from the CW in Tris [13] or in water under heating before [12] and after [22] lipid extraction. These facts allow us to conclude that ncGTGs have different mode of attachment to mGC of CW. However there is no information on the special association of Bgl2 as well as Scw4 molecules into different pools, for example, according to their peculiarities, post-translational modifications (PTMs) or mode of attachment.

There seem to be paradoxes in functioning of mGC-forming ncGTGs: these enzymes have to work on the whole area of the CW and remodel mGC to provide constant growth and division of the yeast cell, at the same time ncGTGs cannot move across mGC of the very rigid CW that is not fluid in contrast to plasma membrane, ncGTGs have to be in close contact with their substrate glucan by being firmly attached to it. Their uncontrolled activity can be hazardous for the cell leading to lytic phenotype caused by a weakened CW. In other words, ncGTGs are constantly sawing off the branch they are sitting on. Therefore, they have to reveal their mGC-forming glucan-remodeling activity only there and then, where and when it is necessary for the cell. It is still unclear how ncGTGs can carry out their functions being localized in the CW, outside of the cytoplasmic membrane on the border of the yeast cell and unstable environment characterized by a variable pH value and ion composition, which are, for example, metabolic factors participating in regulation activity of some enzymes inside the cell.

There is no hypothesis to explain these paradoxes. We hypothesized that the most attractive possible explanation could be different reversible post-translational modifications (PTMs) of ncGTGs’ molecules. Glutathionylation and phosphorylation, for example, are known as reversible modifications that alter or regulate functioning of proteins [23,24]. ncGTGs modified in a different way may have different properties—first of all different affinities for their substrate glucan and therefore have to be attached to it with varying degrees of strength.

In other words, PTMs intended to regulate the crosstalk between ncGTGs and their substrate glucan should be able to reduce or increase the strength of ncGTG attachment to glucan. Revealing of ncGTG pools extractable in different ways characterized by relevant set of PTMs could be an evidence of such correlations.

Such evidence is of a particular interest, as the data on the reversible PTMs of the yeast CW ncGTGs are insufficient in the literature as well as there is no information describing a correlation of PTMs of ncGTGs and the mode of attachment of these enzymes inside the CW.

Another possible explanation may be the location of ncGTGs inside the CW. We supposed that these enzymes have to be compartmentalized in the CW in local zones, for example, for better communications with plasma membrane corresponding microcompartments [25] designated for targeted delivery of excretory vesicles. Such vesicles are migrating through the CW and containing different proteins including modifying enzymes that serve for insertion of reversible PTMs, for instance protein kinases and glutathione-S-transferases [26,27,28].

Up until now there has been almost no information in the literature on the localization of ncGTGs in CW in general and especially the existence of microcompartments containing ncGTGs in CW. Our previously obtained results [12] allow us to assume that at least the major conservative and constitutive yeast CW ncGTG Bgl2 should perform its functions in microcompartments on the entire surface of the cell. Arrangement of glucan-remodeling enzymes in the CW microcompartments could explain a poorly studied mechanism for increasing the surface area of yeast cells during growth similar to the principle of a “tortoise shell”, when individual parts of the surface increase in size along the edges, while the central part does not grow.

In this work we investigated whether the presence of PTMs in ncGTG molecules correlates with differences in strength and/or mode of attachment of these enzymes. We also made our efforts to reveal the localization and peculiarities of ncGTGs in the CW of *S. cerevisiae* with Bgl2 as a model and answer the question of whether it is diffusely distributed in the matrix of this organelle or assembled into microcompartments.

## 2. Results

### 2.1. Non-Covalently Bound GTGs: Different Attachment to CW and PTMs

There are various ways used for extraction of non-covalently bound CW proteins [10,12,13]. In our work at the first stage we investigated the protein content in fractions obtained by sequential extraction of CW with 0.1 M Tris pH 9.8 (T pool) and with water while heating after lipid removal (L pool), because earlier we demonstrated that both ways are efficient for extraction of Bgl2 [13,22]. We also used 6 M guanidine hydrochloride (GuHCl) pH 5.6 (G pool) considering amyloid-like features of some CW proteins, first of all Bgl2.

LC-MS/MS analysis demonstrated that T and L pools were represented by a number of CW mannoproteins, while G pool contained mostly Bgl2. Among ncGTGs we have reliably identified Bgl2, Scw4 and Scw10 in T and L pools. In T pool such proteins as Cwp1, Tos1, Pho3, Eng1 were revealed in addition to these 3 ncGTGs. L pool included Ygp1, Tos1, Pry3, Hsp150, Eng1 in addition to the earlier detected lipid-associated non-covalently bound GPI proteins [22]. Among the last group of proteins in L pool, 3 ncGTGs—Gas1, Gas3 and Gas5—were identified.

We revealed that some of Bgl2 and Scw4 molecules were glutathionylated in T pool: Bgl2 at C68 residue, Scw4 at C354 (Table 1). In T pool we reliably detected three different multiphosphorylated Bgl2 peptides and one multiphosphorylated Scw4 peptide (Table 1) among non-phosphorylated ones. At least two phosphorylated amino acid residues were revealed in different combinations in phosphorylated peptides of Bgl2 or Scw4 in each of the three biological repeats. For example, phosphorylation of Bgl2 was detected in peptides aa^62–101^, aa^99–128^ and aa^224–254^ at 2 or 3 residues and phosphorylation of Scw4 could be detected in peptide aa^339–369^ at 2, 3 or 4 residues in different biological repeats.

In G pool, we identified only glutathione-free Bgl2 molecules and reliably found only one type of Bgl2 phosphorylation at residue T84 (Table 1). This peptide was monophosphorylated in G pool in contrast to multiphosphorylated peptide aa^62–101^ in T pool.

In L pool, Bgl2 was not glutathionylated whereas part of Scw4 peptides was glutathionylated at C173 in contrast to this mannoprotein present in T pool. There were also differences between phosphorylation of Bgl2 and Scw4 molecules in L pool. Bgl2 was not phosphorylated in contrast to Scw4 that was phosphorylated at T137 in L pool and unmodified in T pool (Table 1).

Scw10 in T and L pools was sparsely present and we could not determine glutathionylated or phosphorylated peptides of this ncGTG. We also did not observe stable phosphorylation and glutathionylation of any peptides in Gas1, Gas3 and Gas5. The absence of these PTMs can be explained by the presence of ncGTGs in L pool as an intermediate form in the processing of these proteins before their covalent attachment to the glucan because it is possible to assume that this form does not require regulation with the participation of reversible PTMs.

**Figure 1:**
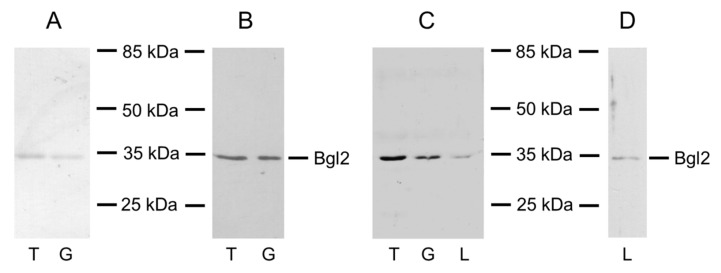
Analysis of extracts from *Saccharomyces cerevisiae* cell walls obtained with 0.1 M Tris for 3.5 h at 30 °C—T, with 6 M GuHCl for 2 h at 30 °C—G (**A**–**C**) and with water at 100 °C after removal of lipid component—L (**C**,**D**). PAGE stained with Coomassie G-250 (**A**) or with silver nitrate staining (**C**) and Western blot stained with antibodies against Bgl2 (**B**,**D**).

We performed PAGE and Western blot analyses to assess the number of major proteins in various pools. By staining with Coomassie G-250, we were able to identify a protein with a molecular weight of about 35 kDa, presented as a major component in T and G pools (Figure 1A) and corresponding to Bgl2. It was confirmed by Western blot analysis with antibodies to Bgl2 (Figure 1B). At the same time, no protein bands were detected in L pool with Coomassie G-250 staining. However, with silver nitrate staining, we were able to identify a protein band not only in T and G pools, but also in L pool (Figure 1C). Western blot analysis confirmed that this protein band was Bgl2 (Figure 1D). This indicates that Bgl2 is also the major protein in L pool.

LC-MS/MS analysis did not give an answer to the question of what site of Bgl2 undergoes N-glycosylation. The peptide containing the putative N202 site was not detected in any of T, G, or L pools. This peptide includes 61 amino acid residues from Q162 to K212 of the Bgl2 sequence. Probably, we could not detect it because of the fact that the peptide was too long. The peptide with the putative site N284 includes 28 amino acid residues from A266 to K293 of the Bgl2 sequence. This peptide was detected in all three pools but without N-glycosylation. It could be supposed that a large molecular mass of this PTM (~2.5 kDa [29,30]) makes it difficult to detect the peptides containing it.

Bioinformatics comparison of Bgl2 orthologs in phylum Saccharomycotina shows that the site of N-glycosylation is conserved at a position corresponding to N202, but not N284 (Supplementary Data 1, Figure S1).

**Figure 2:**
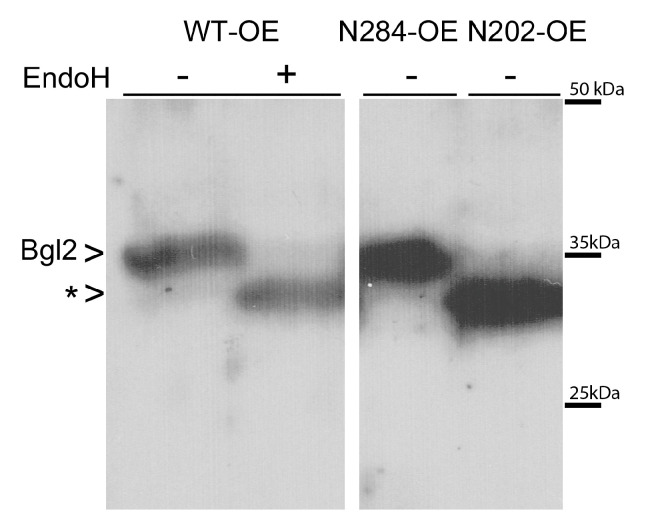
Western blot analysis of *Saccharomyces cerevisiae* cell lysates obtained from WT-OE, N202-OE, and N284-OE strains. Samples were incubated for 15 min with (+) or without (−) endoglycosydase H (EndoH). Bgl2 bands are denoted by arrowheads; (*) indicates unglycosylated Bgl2. Staining with antibodies against Bgl2.

To identify N-glycosylated site we obtained mutations at both potential N-glycosylation sites that either directly replace the asparagine residue in the first N202Q site (strain N202-OE) or replace the serine residue in the second site S286A (strain N284-OE). Analysis of Bgl2 electrophoretic mobility in the extracts obtained by boiling the disrupted cells of the analyzed strains in Laemmli sample buffer provided the following results. The N202Q mutation affected the electrophoretic mobility of Bgl2, and it became similar to the wild-type non-glycosylated (EndoH-treated) Bgl2, the S286A mutation did not affect the electrophoretic mobility of Bgl2 (Figure 2).

The data obtained suggest that the N-glycosylation of Bgl2 in the total preparation occurs only at the N202 site. Therefore, Bgl2 in T, G and L pools should have the same site of N-glycosylation.

### 2.2. Structures Formed by Bgl2 from G Pool

**Figure 3:**
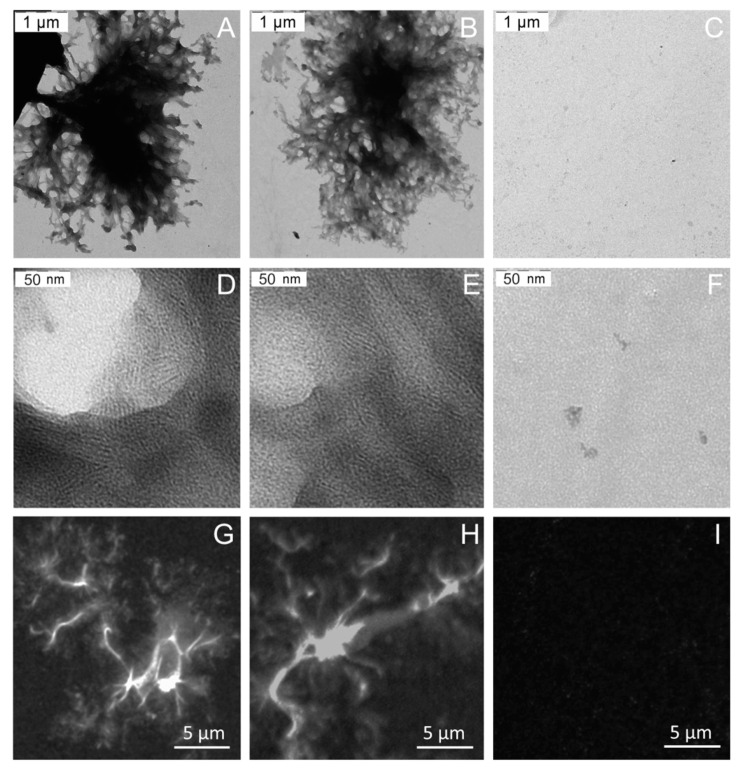
Microscopy of structures formed in G pool extracted from *Saccharomyces cerevisiae* cell walls: TEM (**A**–**F**), immunofluorescence microscopy, staining with antibodies against Bgl2 (**G**–**I**). General view of jellyfish-like associates obtained from *wt* strain (**A**,**B**,**G**,**H**), fibrillar structure of their bodies (**D**,**E**). Control samples from *bgl2Δ* strain (**C**,**F**,**I**).

Because of the fact that we previously demonstrated the ability of Bgl2 to form fibrils after extraction with water after heating [12], we analyzed the extracts of T and G pools with transmission electron microscopy (TEM) to answer the question of what structures could be formed in different pools. In G pool, we observed associates (Figure 3A,B, Supplementary Data 2 and Figure S2A–D). We named them “jellyfish-like” because of their shape and morphology. The bodies of these associates have fibrillar structures (Figure 3D,E, Supplementary Data 2 and Figures S2E,F and S3). Immunofluorescence microscopy allowed us to demonstrate presence of Bgl2 molecules in these structures (Figure 3G,H, Supplementary Data 2 and Figure S4). T pool was presented by an amorphous material (Supplementary Data 2, Figure S5).

We did not analyze the structures formed in L pool, since the preparation of this pool is accompanied by a strong denaturing effect of a chloroform-methanol mixture, and in contrast to GuHCl it is impossible to renature proteins after such treatment.

### 2.3. Bgl2 Molecular Modeling

It is known that PTMs often affect the molecular structure of modified proteins. There was no information earlier on the glutathionylation of Bgl2 in the CW. As this PTM of Bgl2 was identified only in T pool, trying to find correlations between PTM and strength of attachment of ncGTGs in the CW, it was important to analyze whether glutathionylation affect the structure of a Bgl2 molecule.

We carried out a simulation of the structure of Bgl2 with and without glutathione modification of a cysteine residue in position 68 (C68) in the amino acid sequence of Bgl2 to predict the possible role of glutathionylation of Bgl2 molecule. The template for modeling was 4wtp.1 structure of RmBgt17A—a member of glycoside hydrolase family 17 (GH17) from *Rhizomucor miehei* ([31], https://swissmodel.expasy.org/repository/uniprot/P15703).

The degree of similarity of amino acid sequences in Bgl2 and 4wtp.1 was 32.18% that is slightly above the boundary value (30%), which is considered acceptable for the construction of a plausible quality model. Of the 26 amino acid residues forming the cavity containing the active center, 15 amino acid residues, including both the catalytic glutamates E124 and E233, are identical in both structures. The other 7 amino acid residues in Bgl2 are represented by amino acids characterized by similar physical and chemical properties: E→N (35), R→K (61), F→Y (63), Y→W (169), Y→W (281), R→K (282), F→W (295). The remaining 6 amino acid residues do not have similar properties: N→K (34), V→D (67), D→T (92), V→S (168), S→G (232), S→E (275) (Supplementary Data 3, Table S1).

The final models of Bgl2 with and without glutathione molecule were produced by simulation, followed by equilibration of the structure (Supplementary Data 3, Figures S6–S16).

**Figure 4:**
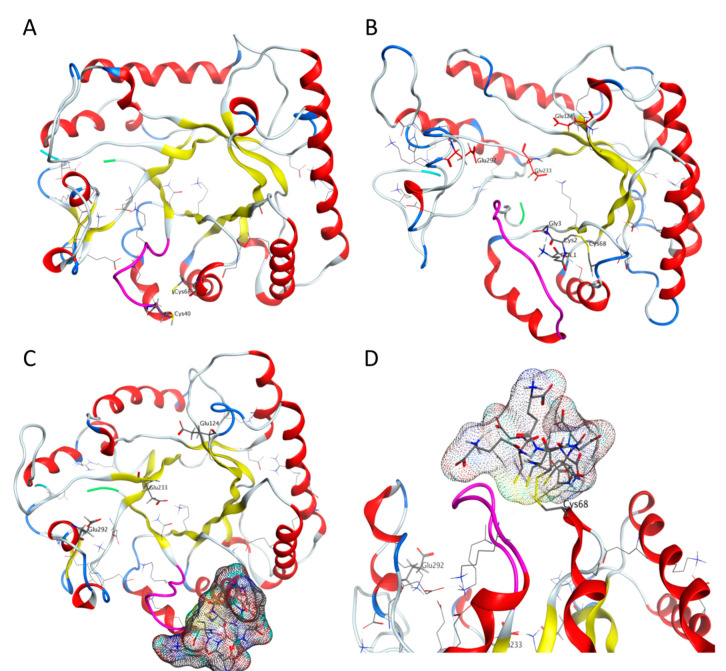
Structural model of Bgl2 molecule without (**A**) and with glutathione (**B**–**D**). N-terminal is highlighted in green, C-terminal is highlighted in cyan. G32-S42 loop is shown in magenta. The glutathione molecule located in the cavity of Bgl2 greatly changes the structure (**B**). The external location of glutathione molecule does not contribute to significant changes in Bgl2 structure (**C**,**D**). Possible conformations of glutathione resulting from molecular dynamics simulation are shown as molecular surface (gray area). Axial (**A**–**C**) and frontal (**D**) views of the cartoon.

Modeling showed that the structure of Bgl2 is relatively stable (Supplementary Data 3, Figures S6–S9). Additional stabilization can be achieved because of the formation of disulfide bonds between cysteine pairs C40-C68 and C262-C310 that are in sufficient proximity to each other (Figure 4A).

Docking of glutathione molecule to Bgl2 structure and molecular dynamics demonstrated that glutathione can be localized both inside (Figure 4B, Supplementary Data 3 and Figures S10–S13) and on the surface (Figure 4C,D, Supplementary Data 3 and Figures S14–S16) of the Bgl2 molecule.

If the glutathione molecule is positioned inside the protein, preferred glutathione location was found to be underneath the G32-S42 loop highlighted in magenta (on Figure 4B glutathione is located to the right of the G32-S42 loop). It leads to significant changes in the structure of Bgl2: a part of the β-barrel structure is disrupted (Figure 4B).

Bgl2 backbone with external location of glutathione as well as Bgl2 molecule without this PTM are stable (Figure 4C,D).

We have applied molecular modeling of phosphorylated Bgl2 to answer two questions: whether single phosphorylation at T84 detected in G pool affects the structure of the Bgl2 molecule and weather the Bgl2 molecule could be phosphorylated simultaneously at all amino acid residues that we have identified as phosphorylated in T pool or not. Modeling has shown that phosphorylation at T84 practically does not change the structure (Supplementary Data 3, Figures S17–21). On the other hand the protein structure is destabilized in the case when the Bgl2 molecule is phosphorylated simultaneously at all detected amino acid residues (Supplementary Data 3, Figures S18, S19, and S22–24). Probably, phosphorylation of all detected residues is unlikely and different Bgl2 molecules have different amino acid residues phosphorylated.

Swiss Model server also has a Scw4 structure, also constructed by homology on the template of 4wtp.1 structure (https://swissmodel.expasy.org/repository/uniprot/P53334). However, sequence identity between Scw4 and 4wtp.1 was only 22.54%. It did not allow us to construct a plausible model for Scw4.

### 2.4. Bgl2 Microcompartments—Mosaic Localization on the Yeast Cell Surface

So far no information has been available on localization of Bgl2 in a yeast CW, and our tasks were first of all to detect this protein on the surface of yeast cells and in their CW and then to verify our hypothesis about localization of Bgl2 in compact microcompartmets. We also performed immunofluorescence microscopy with staining with antibodies against Bgl2 to answer the question of whether the localization of Bgl2 differs in investigated pools.

**Figure 5:**
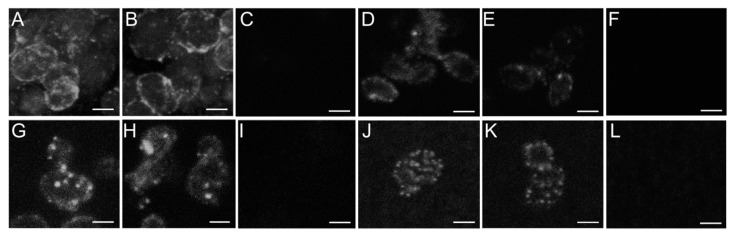
Immunofluorescence microscopy of *Saccharomyces cerevisiae* cells and cell walls stained with antibodies against Bgl2. Scale bars correspond to 2 µm. Cells (**A**–**C**). CW with (**D**,**E**) and without (**F**) EDC crosslinking and boiled in 3% SDS. CW before (**G**–**I**) and after (**J**–**K**) Tris extraction. Control samples from *bgl2Δ* strain (**C**,**I**,**L**).

Up until now all attempts to demonstrate localization of Bgl2 in the CW of *S. cerevisiae* or other yeast species were unsuccessful. However, partial removal of the CW components with glucanase allowed us to visualize Bgl2 (Figure 5). Co-localization of glucanase untreated and treated CW and fluorescent spots achieved by overlay of phase contrast and fluorescent images as well as general view of glucanase treated CW are presented in Supplementary Materials (Supplementary Data 4, Figures S25–S27).

We detected multiple bright individual “patches” (microcompartments) mosaically located on the surface of cells (Figure 5A,B).

To prevent artificial redistribution of Bgl2 in the CW we applied EDC treatment for crosslinking of interacting CW mannoproteins. Bgl2 is visualized as distinctly distinguishable large and small patches in CW after EDC crosslinking (Figure 5D,E). Application of EDC makes it possible to crosslink protein molecules at a distance of a peptide bond. It indicates the close location of mannoproteins in the microcompartments. It should be noted that Bgl2 was not detected in the CW after boiling in 3% SDS if the mannoproteins in the CW were not preliminary crosslinked with EDC (Figure 5F).

Similar localization of Bgl2 was observed in the CW before Tris extraction without crosslinking with EDC (Figure 5G,H). Anti-Bgl2 antibody binding is also noticeable in the area of the inter-spot space of the cells and the CW. After Tris extraction Bgl2 is also visualized, but as multiple and compacted microcompartments without inter-spot space (Figure 5J,K).

The compactly arranged Bgl2 molecules from G pool after isolation from the CW form the structures demonstrated in Figure 3A,B,G,H and in Supplementary Materials (Supplementary Data 2, Figures S2A–D and S3).

We did not reveal Bgl2 in CW after sequential extraction with Tris and GuHCl, despite the fact that these CW must also contain L pool. This may indicate shielding of Bgl2 by the lipid component. Since we were unable to visualize Bgl2, we cannot assert the formation of microcompartments by L pool.

It should be noted that most likely Gas1, maybe Gas3 and/or Gas5 as well, also form microcompartments in the CW (Supplementary Data 5, Figure S28).

### 2.5. Analysis of Lipid Component of CW

The CW is functioning, being in close association with the plasma membrane, despite the fact that these two compartments are separated by the periplasmic space [32]. It was important to determine what is the composition of lipids associated with L pool. Whether the composition of these lipids is typical of the plasma membrane or is significantly different from that. We have investigated the composition of lipids from the CW with thin layer chromatography (TLC). We have found that ceramides, which are typical for rafts of plasma membrane, were not detected even before treatment of the CW preparation with 1% SDS. Other sphingolipids were minorly detected only before SDS treatment of CW. This result may indicate the absence of plasma membrane traces in CW preparations before SDS treatment. Additionally, the absence of a plasma membrane confirms the absence of lipoproteins in L pool. Thus, at this stage of CW purification the lipid rafts as the most resistant to washing components of the plasma membrane are absent. At the same time phospholipids, ergosterol and neutral lipids were found. Quantification of the lipid component of CW is shown in Table 2.

## 3. Discussion

Starting this work, we proceeded from the assertion that the ncGTGs involved in the formation of the new mGC fragments of the CW and in the remodeling of the existing one, are surrounded by their own substrate (mGC glucan) and are unable to move along it.

Being fixed in the matrix of the CW, ncGTGs hydrolyze glucan molecules, insert synthesized de novo oligosaccharides and glycosylated proteins and form mGC complex. Because of their essential functions these enzymes must be either active or inactive in strict accordance with the need. At the same time, mGC is both the object of the action of ncGTGs and a very important structural megamolecule ensuring stability, as well as taking part in dynamic changes of the cell envelope during cell growth, followed by continuous expansion of the CW. Therefore, it is obvious that ncGTGs should be active only when and where it is required.

In order to successfully achieve the co-ordinated work of the ncGTGs ensemble, two circumstances must be met. The first one is the precise regulation and the second is the coordinated control of functioning of ncGTGs localized in the CW. However, there is no recognized hypothesis on the issue.

We hypothesized that reversible modifications of ncGTG molecules may be the best way for such precise regulation. We also suggested that not diffuse, but compact localization of ncGTGs in the CW in local zones is important for the directed and synchronized operation of ncGTGs as it was demonstrated for yeast plasma membrane microcompartments [25].

Compact localization of ncGTGs would also be suitable for the delivery of molecules that can regulate their functioning, for example, enzymes that carry out the reversible PTMs, and are transferred from the cells to the CW, most likely inside vesicles [26,27,28].

The first significant step in testing our hypothesis was answering the question of whether there could be pools of ncGTG molecules with different PTMs in the CW. We also searched for a correlation of presence or absence of such modifications with the properties of ncGTG molecules. First of all, we studied whether the modifications were related to the strength and mode of attachment of ncGTG molecules to the CW.

In this work, we revealed 3 pools of ncGTGs in the CW of *S. cerevisiae* cells that differ in the strength and mode of attachment. We called them “T”, “G” and “L” pools. In T pool as well as in L pool several ncGTGs were detected, but only in Bgl2 and Scw4 peptides were in sufficient quantities so as to allow us to estimate their PTMs (Table 1). These results are supported by the data obtained earlier about predominance of these two ncGTGs in the CW [5,7,8,9,10] and our results (Figure 1 and Table 1). It is important to note that some molecules of Scw4, unlike Bgl2, can covalently attach to mGC and integrate into it [17,18] thereby becoming invisible in our analysis. Other molecules of Scw4 exist in the CW as ncGTG. G pool is represented almost solely by one protein Bgl2 (Table 1). There is a very slim chance that an “invisible” protein, non-digestible by trypsin, is present in G pool besides Bgl2 (according to amino acid sequences from Saccharomyces Genome Database [33], most probably it is not ncGTG). The results obtained indicate that Bgl2 and Scw4 in these three pools have different PTMs (Table 1).

In our work we did not find differences in the extent of N-glycosylation of Bgl2 extracted from the CW under various conditions. N-glycosylation is not considered a reversible modification. It was possible to assume that, while having the same extent of glycosylation (1 oligomannosyl N-glycan per 1 protein molecule), Bgl2 can be represented in the CW by the mix of N202 and N284 glycosylated molecules where N-glycosylation serves as an additional marker for its distribution either into T or G pools (Figure 2). At the beginning of our work, the correlation between the glycosylation sites and distribution of Bgl2 between the pools was unclear because of contradictory N-glycosylation data in the literature [15,16]. In this work, it was shown that in Bgl2 only N202 is glycosylated. It is possible that N-glycosylation of this protein plays a very important role for its attachment to the CW, but this type of PTM is not related to the regulation of its incorporation into T or G pools.

We can conclude that phosphorylation together with a possible presence or absence of association of Bgl2 with other proteins seems to be a discriminating factor for inclusion of Bgl2 into T or G pools. Scw4 is not identified in G pool whereas in T pool multiple phosphorylation is characteristic of its molecules as well as for Bgl2. We suppose that Bgl2 enters the CW as L pool of PTM-free (except N-glycosylation) molecules, it then becomes phosphorylated to a varying extent and forms two pools: multi- and monophosphorylated ones. The first one includes, besides the phosphorylated molecules, the molecules modified and non-modified with glutathione, which are possibly conformationally labile. The second pool, according to the mode of its extraction and data from transmission and fluorescent microscopies, is represented by the molecules tending to fibrillate (Figure 3) and can be approximated by the separate part of Bgl2.

We did not reveal Bgl2 fibrils in T pool where it is highly phosphorylated, as well as in L pool where it is phosphorylated to a small extent. One may say that its ability to fibrillate is affected by the presence of the other proteins because there are many different proteins in L pool (Ygp1, Tos1, Pry3, Hsp150, Eng1 in addition to proteins identified earlier [22]), and some other proteins besides Bgl2 and Scw4 were identified in T pool (Cwp1, Tos1, Pho3, Eng1).

Nevertheless, taking into account a strong denaturing effect that the procedure of obtaining L pool causes, it is possible to consider the role of phosphorylation in the ability of Bgl2 to fibrillate similar to that for Rim4 protein [34].

We found that Bgl2 in T pool contains three peptides with multiple phosphorylation, which probably did not allow Bgl2 from this fraction to demonstrate the ability to form fibrils. Therefore, this protein was extracted in relatively mild conditions. This assumption is also consistent with the fact that Bgl2 extracted in G pool by more rigorous treatment with GuHCl capable of dissolving amyloid proteins [35,36,37] has only one phosphorylated peptide with one phosphorylated amino acid residue.

We also suppose that Scw4 enters the CW as a part of L pool with both modifications: C173-glutathionylated and T137-phosphorylated molecules, as well as without these modifications. At the same time, according to the literature there are two pools of Scw4 molecules in the CW that are covalently and non-covalently attached to glucan [17,18]. In our analysis we did not reveal Scw4 molecules being C173-glutathionylated and T137-phosphorylated in T pool. However, in T pool we revealed differently modified Scw4: C354-glutathionylated and multiphosphorylated molecules (Table 1). The results obtained may suggest that different reversible modifications that we revealed for Scw4 as well as for Bgl2 may serve as a marker of their distribution in different pools.

Our results allow us to suggest that the lipid component in the CW is very similar to lipid droplets, previously described in yeast [38,39]. The outer layer of the lipid droplets is represented by phospholipids, the middle layer by ergosterol, and the inner layer contains neutral lipids [39]. We identified all components of the lipid droplets in the CW before SDS treatment (Table 2). It should be noted that the CW preparations before SDS treatment did not contain plasma membrane traces. After washing with SDS, phospholipids practically disappear from the CW, but ergosterol and neutral lipids remain (Table 2). It is possible to suppose that treatment with SDS could lead to the removal of an outer layer of the lipid droplets.

Lipid droplets have been shown to be essential for the process of CW biogenesis in sporulating yeast [40]. It is possible that lipid droplets also play an important role in the functioning of the CW of a vegetative cell. It is possible that lipid droplets are a lipid component that is associated with ncGTG, identified as L pool in this work.

We revealed that part of Bgl2 and Scw4 extracted in T pool had glutationylated C68 and C354 in their molecules respectively.

Glutathionylation plays an important role in a number of metabolic, signaling, and transcription processes, modulating the functioning of the proteins involved in these processes and protecting them from oxidative stress [41]. However, relatively small amounts of glutathionylated proteins were found in bacteria and yeast compared to mammalian cells [42]. C68 glutathionylation was reliably detected in some Bgl2 molecules in T pool, and the absence of this modification of C68 in Bgl2 in G pool may indicate different functions of these two pools of this protein. It is possible that Bgl2 obtained by extraction in Tris may undergo glutathionylation in response to oxidative stress or may be constitutive [41]. The latter could be realized for protein–protein interaction and for regulation of enzymatic activity [23,41,42].

We believe that such a modification may be important for the functioning of Bgl2, for example, it can stimulate or inhibit the interaction of Bgl2 molecules with each other or with other CW proteins. To understand the structural basis of the observed differences in the properties of Bgl2 from T and G pools, we simulated the structure of Bgl2 with and without glutathione (Figure 4).

If the glutathione molecule is located inside the protein, significant changes in the structure of Bgl2 occur (Figure 4A,B), part of the structure of the β-barrel is disrupted. The C-terminal portion of the Bgl2 molecule with the glutathione facing inwards is modified and becomes much more conformationally labile. This does not allow us to conclusively confirm the possibility of formation of a disulfide bond between C262 and C310 residues. Such an arrangement of glutathione with high probability can strongly influence the arrangement of amino acid residues in the zone of the active center of the enzyme or in the zone of its binding to the substrate and affect the activity of Bgl2. It was found that the Bgl2 fold with the external arrangement of glutathione is stable, as well as the Bgl2 molecule without this PTM (Figure 4A,C,D). However, in this case, the formation of a disulfide bond between the C262 and C310 is definitely more difficult, since C262 is located inside the molecule, and this prevents C310 from approaching it. Probably the conformational mobility of the C-terminus plays a role in the formation of Bgl2 oligomers capable of enzymatic activity.

In our work we visualized Bgl2 on the surface of the whole cells, as well as in the isolated CW both with and without crosslinking of CW mannoproteins (Figure 5). It is important to note that there has been no information available on the localization of this ncGTG in yeast CW until now.

It was crucially important that we identified Bgl2-zones in the CW, which we named microcompartments or patches, not only in isolated *wt* cells (Figure 5A,B), but also in the CW even after treatment of yeast cells with EDC crosslinker (Figure 5D,E) that is able to connect protein molecules at a peptide bond distance. This assured us that consequent procedures aimed at visualization of these proteins did not affect their native localization. This fact allowed us to believe in the other results of visualization of the patches obtained without EDC pretreatment (Figure 5G,H,J,K).

Using Bgl2 as an example, we showed that ncGTGs can be located irregularly on the cell surface: in separate patches (microcompartments). The presence of Bgl2 in large and small compact patches with inter-spot space before (Figure 5D,E,G,H) and only small patches (in a larger amount on the CW) after (Figure 5J,K) T pool extraction allows us to suppose that Bgl2 from G pool could be shielded by Bgl2 from T pool. Moreover, Bgl2 from G pool could be inside T pool.

The absence of possibility of lateral movement along the CW and abundant presence in close contact with its substrate—glucan of the mGC require special conditions of activity regulation and both serve as an evidence of rigid fixation of Bgl2 inside the CW.

In this work, we obtained convincing confirmation of the previously discovered fact that at least part of Bgl2 may be present in the CW in the form of molecules with a strong tendency to fibrillation. This part of the protein is extracted into GuHCl and, after extraction, tends to associate into fibrils.

**Figure 6:**
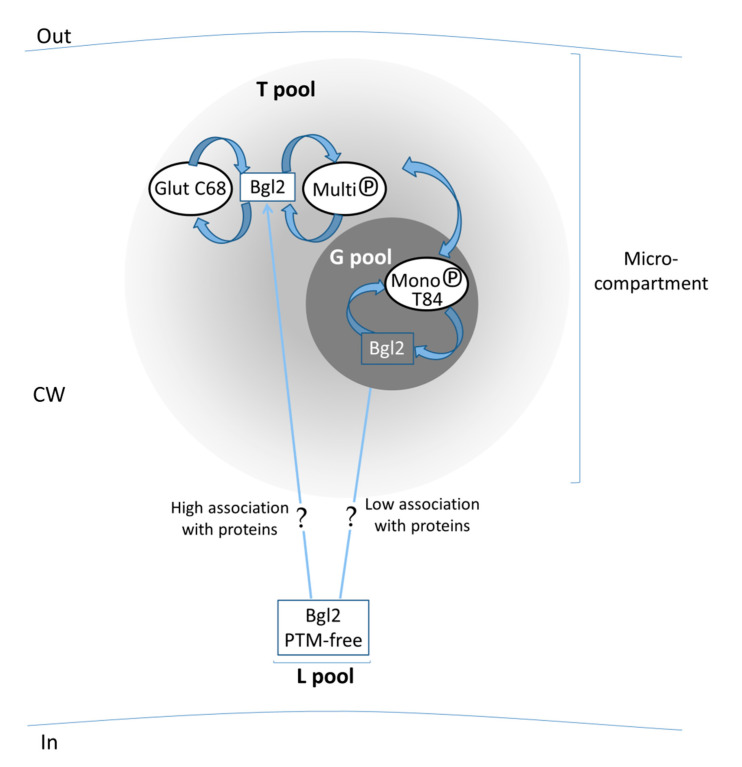
The hypothetical scheme of *Saccharomyces cerevisiae* cell wall segment with a microcompartment. We suppose that PTM-free Bgl2 enters the cell wall as a part of L pool. Depending on the degree of association with proteins in L pool, Bgl2 is directed to T or G pool. Probably, Bgl2, acquiring one or another set of PTMs, can migrate between T and G pools. In these two pools, at least some of the molecules are closely located, therefore, we suppose that they form the microcompartment. The localization of L pool could not be revealed. It is possible that proteins of L pool are dispersed rather than compactly located in the cell wall. Glut—glutathionylation, MultiP and MonoP—multi- and monophosphorylation, respectively.

In Figure 6 we summarize our results based on the revealed correlations between the different PTMs and the strength/mode of attachment of Bgl2 in the CW also taking into consideration the results obtained in the experiments on visualization of the location of this ncGTG inside the CW.

The mosaic arrangement of GTGs (first of all, Bgl2) suggests that the growth and extension of the rigid lateral CW can be carried out according to the principle of growth of the tortoise shell, when the entire surface as a whole increases its area by increasing the area of individual sections while most of its surface remains unchanged. Our results give the first evidence of a mosaic arrangement of GTGs in the CW of yeast and reveal multiple post-translational modifications that correlate with their properties, primarily with the strength of attachment of GTGs in the CW. Together, these data confirm our assumptions and may be the key to further studies leading to an answer to the question of how the mGC remodeling enzymes ncGTGs function.

At the beginning of our experimental work, the mode of attachment and regulation of functioning of non-covalently bound glucanosyltransglycosylases of *Saccharomyces cerevisiae* CW seemed to be an unsolved multipronged puzzle. We made assumptions regarding the key points—PTMs, localization, and attachment of these enzymes—and concentrated our efforts on that. We obtained the data that allow to fill being summarized with results obtained earlier [5,6,7,8,9,10,18,22,43] in a part of the puzzle and are helpful in our planning further scientific investigations, namely, discovering the role of each modification for the yeast cells under different conditions, or whether enzymes can change localization during cell growth and division, as well as under stress, and, as well, whether other enzymes within the CW are arranged in microcompartments or not.

## 4. Materials and Methods

*S. cerevisiae* strains used in this work are presented in Table 3.

### 4.1. Construction of Yeast Strains

To obtain potential Bgl2 N-glycosylation site mutants, PCR mutagenesis of plasmid-encoded *BGL2* gene [7] was performed with Q5 site-directed mutagenesis kit (New England Biolabs, Moscow, Russia) according to manufacturer’s instructions. For construction of pBGL2-N202 plasmid with N202Q mutation 5′-CAAGGTCAAACCATGCAACAAGCTTCTTACTCATTCTTTGATGATATTATGC-3′ and 5′-CCAGTAGGAGAACGCGTTAG-3′ primers were used. For construction of pBGL2-N284 plasmid with S286A mutation 5′-GAAGATTGGAAGCCAAACACTGCAGGTACCTCTGATGTCG-AGAAG-3′ and 5′-ATCAAAGGCTTCAAAAACAATAAC-3′ primers were used. Obtained plasmids pBGL2-N202 and pBGL2-N284 were verified by sequencing and transformed into *bgl2Δ* strain to produce N202-OE and N284-OE strains respectively.

### 4.2. Yeast Growth Conditions

BY4742 yeast strain was grown in liquid nutritious YPD medium (1% yeast extract, 2% peptone, 2% glucose). WT-OE, N202-OE, and N284-OE yeast strains were grown in an uracil-free synthetic medium [45] supplemented with 2% D-galactose for the induction of *BGL2* under the control of the GAL10-CYC1 hybrid promoter. The cell cultures were grown for 19 h (log-phase) at 30 °C with agitation on an orbital shaker (New Brunswick, Moscow, Russia) at 200 rpm.

### 4.3. Yeast CW Isolation

Log-phase yeast cells were precipitated by centrifugation for 10 min at 1650× *g* (Rotina, Moscow, Russia), washed twice with 0.05 M potassium-phosphate buffer pH 8.0 and disrupted in the shaker (Heidolph, Moscow, Russia) with glass beads (0.5 mm; Sigma, Moscow, Russia) under cooling. The extent of cell disruption was estimated with a light microscope (Opton, Moscow, Russia). CW preparations containing less than 0.1% of intact cells were used in the further work. CW were separated from the intracellular content by centrifugation at 2580× *g* for 5 min. CW and cells formed the double-layer precipitate; CW forming the upper layer were carefully suspended in water and separated from the cells. CW were washed twice with water, twice with 1% sucrose, twice with 1 M NaCl, twice with 1% NaCl, and once with water. The amount of CW was estimated spectrophotometrically (absorbance at 540 nm, A540) [13]. CW obtained by this procedure will be further named “untreated”.

### 4.4. Yeast CW Partial Deproteinization

For a partial deproteinization CW were treated with 1% SDS for 1 h at 37 °C. Deproteinized CW were separated by centrifugation at 2580× *g* for 5 min and were washed five times with 0.2 M Na-Ac buffer pH 5.6, three times with n-butanol-water mixture 0.7:1 (*v*/*v*) and with water to remove traces of SDS [13]. CW obtained by this procedure will be further named “purified”.

### 4.5. Glucanase Treatment of Cells and CW

Log-phase yeast cells were precipitated, washed twice with water, and incubated in a 3.7% paraformaldehyde in 1× PBS solution pH 7.4 for 20 min at room temperature and then overnight at 4 °C. Fixed cells were washed twice with 50 mM NH_4_Cl in 1× PBS solution pH 7.4 and twice with 1× PBS solution pH 7.4. After that yeast cells were treated with β-1,3-glucanase from *Hordeum vulgare* (Megazyme, Moscow, Russia) in 1.2 M mannitol for 4 h at 37 °C (in ratio 12.5 units of β-1,3-glucanase activity to 100 optical units of yeast cells A540), followed by washing twice with 1.2 M mannitol solution to remove β-1,3-glucanase and then were used as a preparation for immunofluorescence microscopy.

Purified CW were washed twice with 50 mM NH_4_Cl in 1× PBS solution pH 7.4 and twice with 1× PBS solution pH 7.4. Then CW were treated with β-1,3-glucanase from *Hordeum vulgare* (the same ratio) in water for 4 h at 37 °C, followed by twice washing with water to remove β-1,3-glucanase and then were used as a preparation for immunofluorescence microscopy.

### 4.6. CW Protein Crosslinking

Untreated CW were processed with 0.1% SDS for 15 min at room temperature, washed five times with 0.2 M Na-Ac buffer pH 5.6 and then with water. Crosslinker EDC (1-ethyl-3-(3-dimethylaminopropyl)carbodiimide hydrochloride) (ThermoFisher Scientific, Moscow, Russia) was added to CW transferred to MES buffer in the ratio 1 optical unit CW (A540) in 10 µL buffer to the final concentration 2.5 or 5 mM. Obtained mixture was incubated for 2 h at room temperature. Then CW were separated by centrifugation and washed with water three times. Uncrosslinked CW proteins were removed with 3% SDS under boiling for 10 min. CW were washed five times with 0.2 M Na-Ac buffer pH 5.6 and with water. Obtained CW were treated with β-1,3-glucanase from *Trichoderma* sp. (Megazyme, Moscow, Russia) overnight at 37 °C (in ratio 0.9 units of β-1,3-glucanase activity to 100 optical units of yeast cells A540), followed by numerous washing off β-1,3-glucanase with water and then were used as a preparation for immunofluorescence microscopy.

### 4.7. Sequential Extraction of Non-Covalently Attached CW Proteins

#### 4.7.1. Extraction of T Pool

Purified CW in a ratio of 1 optical unit (*A_540_*) to 10 µL of 0.1 M Tris solution pH 9.8 were incubated for 3.5 h at 30 °C. The extract was separated from the CW by centrifugation at 12,000× *g* (Eppendorf, Moscow, Russia) for 2 min.

#### 4.7.2. Extraction of G Pool

CW after Tris extraction were washed with 0.1 M Tris twice and with Milli-Q H_2_O three times. Then CW were incubated in 6 M GuHCl pH 5.6 in a ratio of 1 optical unit of CW (A540) to 10 µL of 6 M GuHCl for 2 h at 30 °C and agitation 200 rpm. The extract was separated from the CW by centrifugation at 12,000× *g* for 5 min and dialyzed against water overnight. Dialysis tubes with a cut-off limit of 6–8 kDa (Serva, Moscow, Russia) were prepared by boiling in 10 mM EDTA (Sigma, Moscow, Russia) and then in water.

Tris extract was neutralized with 1.2 M Na-Ac buffer pH 5.6 to final molarity and pH of solution 0.8 M and 5.6, respectively. Tris and GuHCl extracts from CW of *wt* and *bgl2Δ* strains were treated with β-1,3-glucanase (0.125 units to 100 µL of extracts) from *Hordeum vulgare* (Megazyme, Moscow, Russia) overnight at 4 °C before immunofluorescence microscopy and for 7 h at 30 °C before TEM.

#### 4.7.3. Extraction of L Pool

CW extracted with GuHCl were washed 8 times with Milli-Q H_2_O and centrifuged for 5 min at 12,000× *g*. Chloroform-methanol mixture (2:1; *v*/*v*) was added to the pellet of CW (20 optical units of CW A540 in 1 mL of mixture), thoroughly mixed and incubated for 1 h at 30 °C and agitation 200 rpm and then centrifuged at 12,000× *g*, according to Bligh and Dyer [46] with modifications. CW were washed with water until complete disappearance of chloroform in the supernatant. Proteins were extracted by adding Milli-Q H_2_O (10 µL per 1 optical unit of CW A540) and heating at 100 °C for 5 min. The extracts were analyzed by LC-MS/MS.

### 4.8. Preparation of Cell Lysates

Cells of overexpressing strains WT-OE, N202-OE, and N284-OE were disrupted as described in the section “Yeast CW isolation.” Obtained preparations (cell lysates) of disrupted cells were equilibrated by total protein concentration according to Lowry [47] with modifications. Samples were analyzed by polyacrylamide gel electrophoresis (PAGE) with and without endoglycosidase H (EndoH, Sigma, Moscow, Russia) treatment. Incubation with EndoH was carried out for 15 min at 37 °C in the ratio 0.0004 units of EndoH activity to 20 µL of sample.

### 4.9. Electrophoresis, Western Blot Analysis

PAGE was performed according to Laemmli [48] with modifications (Laemmli buffer additionally contained 5% β-mercaptoethanol and 0.625 mM EDTA) in 4% concentrating and 12% resolving polyacrylamide gel [13]. Various CW extracts were equalized by the optical density of CW at 540 nm. Cell lysates were equalized by the amount of proteins according to Lowry [47]. PAGE was performed in the presence of prestained protein molecular weight markers (Fermentas, Moscow, Russia). Protein staining in gel was performed with Coomassie G-250 according to Peisker [49] or with silver nitrate according to Gharahdaghi [50] with modifications. To identify Bgl2 bands Western Blot analysis was performed according to Rekstina [22] with modifications. Primary polyclonal antibodies against Bgl2 were raised in male BALB/c mice (SPF status) in the laboratory of Dr. O.S. Morenkov (Institute of Cell Biophysics, Russian Academy of Sciences, Pushchino, Russia) with PAGE-purified protein (40 μg per mouse) and were used in previous investigations [12,13,22]. Secondary polyclonal rabbit anti-mouse IgG antibodies were labeled by horse radish peroxidase (Invitrogen, Moscow, Russia). Protein–antibody complexes were visualized by enhanced chemiluminescence using the ThermoFisher Scientific ECL system (ThermoFisher Scientific, Moscow, Russia).

### 4.10. LC-MS/MS Analysis

Sample preparation was performed as described previously with minor modifications [51]. Sodium deoxycholate (SDC) reduction and alkylation buffer pH 8.5 were added to extracts containing 100 µg protein so that the final concentration of protein, Tris, SDC, TCEP, and 2-chloroacetamide were 1mg/mL, 100 mM, 1% (*w*/*v*), 10 mM, and 40 mM, respectively. This stage was omitted for samples that were prepared for the determination of post-translational modifications.

The solution was boiled for 10 min and after cooling down to room temperature, the equal volume of trypsin solution in 100 mM Tris-HCl pH 8.5 was added in a 1:100 (*w*/*v*) ratio. Digestion was performed at 37 °C overnight. Peptides were acidified to a final concentration of 1% trifluoroacetic acid (TFA) for SDB-RPS binding, and 20 µg was loaded on two 14-gauge StageTip plugs. Ethylacetate/1% TFA (125 mL) was added, and the StageTips were centrifuged at 200× *g*. After washing the StageTips using two wash steps of 100 µL ethylacetate/1% TFA and one of 100 µL 0.2% TFA consecutively, peptides were eluted by 60 µL of elution buffer (80% acetonitrile, 5% ammonia). The collected material was completely dried using a SpeedVac centrifuge (Savant, Moscow, Russia) and stored at −80 °C before LC-MS/MS analyses. Before analyses peptides were suspended in loading buffer (2% acetonitrile, 0.1% TFA) and sonicated for 2 min (Elmasonic S100, Elma, Moscow, Russia).

Approximately 1 µg of peptides was loaded for 2 h gradient. Peptides were separated on a 25-cm 75-µm inner diameter column packed in-house with Aeris Peptide XB-C18 2.6 µm resin (Phenomenex, Moscow, Russia). Reverse-phase chromatography was performed with an Ultimate 3000 Nano LC System (ThermoFisher Scientific, Moscow, Russia), which was coupled to the Q Exactive HF mass spectrometer (ThermoFisher Scientific, Moscow, Russia) via a nanoelectrospray source (ThermoFisher Scientific, Moscow, Russia). Peptides were loaded in buffer A (0.2% (*v*/*v*) formic acid) and eluted with a linear 120-min gradient of 4–45% buffer B (0.1% (*v*/*v*) formic acid, 80% (*v*/*v*) acetonitrile) at a flow rate of 350 nL/min. After each gradient, the column was washed with 95% buffer B for 5 min and reequilibrated with buffer A for 5 min. Column temperature was kept at 40 °C. Peptides were analyzed on a mass spectrometer, with one full scan (300–1400 *m*/*z*, R = 60,000 at 200 *m*/*z*) at a target of 3 × 10^6^ ions, followed by up to 15 data-dependent MS/MS scans with higher-energy collisional dissociation (HCD) (target 10^5^ ions, max ion fill time 60 ms, isolation window 1.4 *m*/*z*, normalized collision energy (NCE) 28%, underfill ratio 2%), detected in the Orbitrap (R = 15,000 at fixed first mass 100 *m*/*z*). Other settings: charge exclusion—unassigned, 1, >6; peptide match—preferred; exclude isotopes—on; dynamic exclusion—30 s was enabled. Each sample was analyzed by LC-MS/MS in the three biologic replicates.

### 4.11. Immunofluorescence Microscopy of Samples Stained with Antibodies

Images of yeast cells and CW treated in different ways were obtained with fluorescent confocal scanning microscope Leica TCS SP2 AOBS (Leica, Rostock, Germany). Images of material from protein extracts (T and G pools) were obtained with fluorescent confocal scanning microscope Carl Zeiss Axiovert 200M LSM 510 META (Zeiss, Moscow, Russia).

Yeast cells, CW, and protein extracts (T and G pools) were fixed on glass slides with 3.7% paraformaldehyde for 20 min at room temperature incubated in conditions preventing desiccation. Samples were stained with mouse primary polyclonal antibodies against Bgl2 (obtained as described in the Section 4.9) and secondary polyclonal goat anti-mouse antibodies IgG labeled with Alexa-488 fluorophore (Invitrogen, Moscow, Russia) or with rabbit primary polyclonal antibodies against Gas1 and secondary polyclonal goat anti-rabbit antibodies IgG labeled with Alexa-647 fluorophore (Invitrogen, Moscow, Russia; Rostock, Germany). Antibodies to Gas1 were kindly provided by Dr. M.O. Agaphonov (Federal Research Center “Fundamentals of Biotechnology”, Russian Academy of Sciences, Moscow, Russia).

Cells, CW, and protein extracts of *bgl2Δ* strain were used as the antibody controls to cells and CW of *wt* strain. Untreated with EDC CW of *wt* strain were used as the control to EDC-crosslinked CW of *wt* strain.

### 4.12. Transmission Electron Microscopy (TEM)

Small volumes (2 µL) of protein extracts (T and G pools) from CW of *wt* and *bgl2Δ* strains were absorbed onto glow-discharged carbon-coated, Formvar-filmed 200-mesh copper grids overnight in conditions preventing desiccation. Negative-staining with 2% uranyl acetate solution was performed for 2 min. Grids were allowed to dry in a light-protected environment and were visualized on electron microscopes JEM-100B or JEM-1011 (JEOL, Moscow, Russia).

### 4.13. Determination of CW Lipids

Extraction of lipids from CW treated or untreated with SDS was performed according to Bligh and Dyer [46] with modifications. CW were incubated in the mixture of chloroform-methanol mixture (2:1; *v*/*v*) for 2 h at 30 °C and 200 rpm. Obtained extract was separated from the CW by centrifugation at 12,000× *g* (Minispin, Moscow, Russia) for 10 min. The pellet was incubated once more in the water-chloroform-methanol mixture (0.8:1:2; *v*/*v*/*v*) and centrifuged. Both supernatants were combined. Chloroform and water in equal volumes were added to the total supernatant until the ratio of water-chloroform-methanol mixture (1.8:2:2; *v*/*v*/*v*). The obtained mixture was intensively shaken and centrifuged. The lower chloroform phase was separated and evaporated. For removal of protein impurities, the resulting lipid fraction was dissolved in a small volume of benzene and centrifuged. The benzene fraction was evaporated and the resulting extract containing lipids was analyzed for the presence of phospholipids and neutral lipids.

Separation of phospholipids and neutral lipids was carried out by the method of thin layer chromatography (TLC) on glass plates for high-performance thin layer chromatography (HP-TLC) 10 × 10 and 10 × 5 cm with a fixed layer of silica gel (Merck, Moscow, Russia). Chromatography of phospholipids was carried out in a chloroform-methanol-formic acid solvent system (65:25:4, *v*/*v*/*v*). Neutral lipids were identified in a solvent system: hexane-diethyl ether-acetic acid (80:20:1, *v*/*v*/*v*). A double solvent system was used to separate the ceramides: chromatography was first carried out in diethyl ether and then in a chloroform-methanol-water system (40:10:1, *v*/*v*/*v*) 2/3 of the initial height. Phospholipids, ceramides, and neutral lipids (Sigma, Moscow, Russia) were used as standards.

Samples were applied as dots with a micro-syringe in volumes from 2 μL to 100 μL (lipid concentration in the mixture from ~2.5 mg/mL to ~5 mg/mL). After chromatography, the plates were dried. To identify spots on the plates, they were stained with 10% CuSO4 solution and 8% orthophosphoric acid. Then the plates were incubated at 160 °C during 10 min.

The amount of detected lipids was analyzed with Image J software. The spots corresponding to each detected lipid were calculated by intensity of their brightness. The amount of lipids was statistically estimated. The confidence interval was calculated using the Student’s *t*-test.

### 4.14. Bioinformatic Analysis

The structure of the Bgl2 molecule built by the Swiss Model server [52] on 4wtp.1 template was used (https://swissmodel.expasy.org/repository/uniprot/P15703).

Initial Bgl2 model was placed into dodecahedral unit cell for use with periodic boundary conditions (cell parameters a = b = c = 64 Å, α = β = 60°, γ = 90°), solvated with TIP3P water [53] and ionized with NaCl to 0.12M. Total energy was minimized for 5000 steps using steepest descent minimizer, then the system was equilibrated, with protein heavy atoms harmonically constrained, for 50,000 steps in NVT regime using Berendsen thermostat, followed by NPT equilibration for 500,000 steps. During the production run, temperature was controlled by Nose-Hoover method [54] and pressure by isotropic Parrinello-Rahman coupling method [55]. Temperature setpoint was 310 K. Data frames were recorded every 100 ps. Timestep used for equilibration and production was 2fs with LINKS method of H-bonds constraining. Total of 100 ns production trajectory was obtained.

Bgl2-glutathione conjugate model was built by docking. Glutathione conformations were clustered using DBSCAN method, and the most populated cluster was selected. Steered molecular dynamics was applied to assess the possibility of glutathione folding “inside” the Bgl2. Additional harmonic potential was applied to the glutathione center of mass to assess.

All simulations were performed with full-atom CHARMM36 force field [56], using GROMACS software [57] on 6-core 3.50GHz Intel(R) Xeon(R) CPU E5-1650 v3 workstation equipped with dual Quadro K2200 GPUs (Nvidia). VMD [58] was used for MD trajectory analysis and visualization.

## Figures and Tables

**Table 1:** Post-translational modifications of Bgl2 and Scw4 in T, G and L pools. Glut—glutathionylation, P—phosphorylation. Modified amino acid residues are underlined and highlighted in bold.

Pool		Modified Peptides of ncGTGs	
	PTM Type	Bgl2	Scw4
**T**	Glut	^62^VYAASD**C**NTLQNLGPAAEAEG FTIFVGVWPTDDSHYAAEK^101^	^345^DAVSAITSS**C**GADT FLFTAFNDYWK^369^
	P	^62^VYAA**S**DCN**T**LQNLGPAAEAEGF**T** IFVGVWPTDDSHYAAEK^101^	^339^**S**KENQKDAV**S**AI**TSS**C GAD**T**FLFTAFNDYWK^369^
		^99^AEKAALQ**TY**LPKIKE**S** TVAGFLVGSEALYR^128^	
		^224^**ST**DI**T**FWVGE**T**GWPTDG **T**NFESSYPSVDNAK^254^	
**G**	P	^62^VYAASDCNTLQNLGPAAEAEGF **T**IFVGVWPTDDSHYAAEK^101^	^-^
**L**	Glut	-	^168^LYGTD**C**NQVENVFK^181^
	P		^135^GI**T**YTPYESSGACK^148^

**Table 2:** Quantification of lipid component in *Saccharomyces cerevisiae* cell walls before and after SDS treatment**.**

Revealed Lipids		CW Before SDS Treatment (µg of Lipids in 10 Optical Units of CW)	CW After SDS Treatment (µg of Lipids in 10 Optical Units of CW)
Phospholipids	Sphingomyelin	0.58 ± 0.16	-
	Phosphatidylcholine	1.82 ± 0.78	-
	Phosphatidylethanolamine	0.96 ± 0.15	0.92 ± 0.33
	Ergosterol	1.98 ± 0.43	1.36 ± 0.24
Neutral lipids	Fatty acids	33.60 ± 4.30	34.22 ± 3.05
	Methyl esters of fatty acids	12.73 ± 2.75	11.52 ± 2.46
	Triglycerides	4.59 ± 0.29	3.68 ± 0.43
	1,3-Diglycerides	1.18 ± 0.41	1.13 ± 0.04

**Table 3:** *Saccharomyces cerevisiae* strains used in present research.

Strain	Genotype	Source
BY4742 (*wt*)	MATα *his3Δ1 lys2Δ0 ura3Δ0 leu2Δ0*	EUROSCARF [44]
*bgl2Δ*	MATα *his3Δ1 lys2Δ0 ura3Δ0 leu2Δ0 bgl2::LEU2*	[7]
WT-OE *	MATα *his3Δ1 lys2Δ0 ura3Δ0 leu2Δ0 bgl2::LEU2 pEMBLyex4 [URA3 dLEU2 P_GAL10-CYC1_-BGL2]*	[7]
N202-OE *	MATα *his3Δ1 lys2Δ0 ura3Δ0 leu2Δ0 bgl2::LEU2 pEMBLyex4 [URA3 dLEU2 P_GAL10-CYC1_-BGL2-N202Q]*	Present work
N284-OE *	MATα *his3Δ1 lys2Δ0 ura3Δ0 leu2Δ0 bgl2::LEU2 pEMBLyex4 [URA3 dLEU2 P_GAL10-CYC1_-BGL2-S286A]*	Present work

* Overexpressing strains.

## Supplementary Materials

Supplementary materials can be found at https://www.mdpi.com/1422-0067/21/21/8304/s1.

## Abbreviations

CW	Cell walls
EDC	1-Ethyl-3-(3-Dimethylaminopropyl)carbodiimide hydrochloride
EndoH	Endoglycosidase H
G pool	Guanidine hydrochloride fraction of CW proteins
GuHCl	Guanidine hydrochloride
GPI	GlycosylPhosphatidylInositol
GTGs	Glucanosyltransglycosylases
L pool	Lipid-associated fraction of CW proteins
mGC	mega-GlycoConjugate
ncGTGs	Non-covalently bound glucanosyltransglycosylases
PAGE	Polyacrylamide Gel electrophoresis
PTM	Post-translational modification
SDS	Sodium dodecyl sulfate
T pool	Tris fraction of CW proteins
TEM	Transmission electron microscopy
TLC	Thin layer chromatography
YPD	Yeast, peptone, D-glucose
