# Supplementary material for: The Post-Translational Modifications, Localization, and Mode of Attachment of Non-Covalently Bound Glucanosyltransglycosylases of Yeast Cell Wall as a Key to Understanding their Functioning
Source: Int J Mol Sci. 2020 Nov 5;21(21):8304. doi: 10.3390/ijms21218304 (PMC7663962; doi:10.3390/ijms21218304)
Supplement: Supplementary file 1 [file ijms-21-08304-s001.pdf]

Supplementary Materials

Supplementary Data 1

CW mannoproteins that are similar to Bgl2 molecular weight and single site N-glycosylation (~2.5 kDa) were detected in CW of *Kluyveromyces lactis* [29], *Candida albicans*, *Hansenula wingei* (=*Wickerhamomyces canadensis*), *Pichia scolyti* (=*Yamadazyma scolyti*), *Saccharomycopsis lypolytica* (=*Yarrowia lipolytica*) [30] and are expected to correspond to Bgl2 orthologs. Mannoprotein gp29 in *K. lactis* was detected with antibodies against *S. cerevisiae* gp29(=Bgl2) [29]. Some of the orthologs of Bgl2 (including *C. albicans*, *K. lactis*, *W. canadensis* and *Y. lipolytica*), have a single N-glycosylation sequon that corresponds to N202 site in *S. cerevisiae* Bgl2. *Y. scolyti* Bgl2 ortholog has two N-glycosylation sequons that correspond to *Sc*Bgl2 K110 and N202 positions. Thus, we would expect that only N202 site has N-glycosylation based on literature data on possible Bgl2 orthologs. By applying bioinformatic tools we further extended our analysis to the conservation of N202 and N284 potential N-glycosylation sites among the known and putative Bgl2 orthologs in phylum Saccharomycotina. Results of this analysis are presented in Figure S1, from that we also expect that only N202-site is normally N-glycosylated.

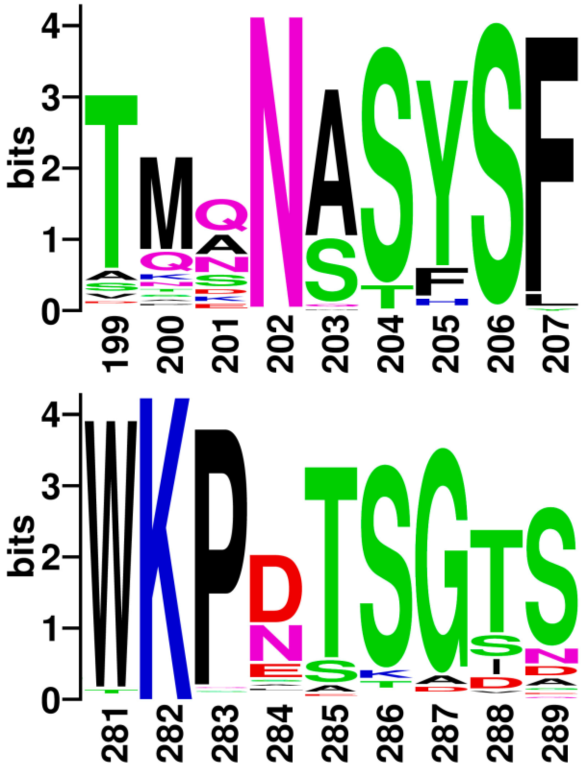

Figure S1. Sequence logos of *Saccharomyces cerevisiae* Bgl2 orthologs from phylum Saccharomycotina for regions corresponding to N202 and N284 potential N-glycosylation sites of *Sc*Bgl2. Protein sequences were found by NCBI-tblastn search in genomes of phylum Saccharomycotina with e-value threshold 1e-50 and lower. Multiple sequence alignment was produced using Clustal Omega server with default parameters. Sequence logos were produced using WebLogo server. N-glycosylation sequon (N-x-S/T) is conserved only at N202 position.

Supplementary Data 2

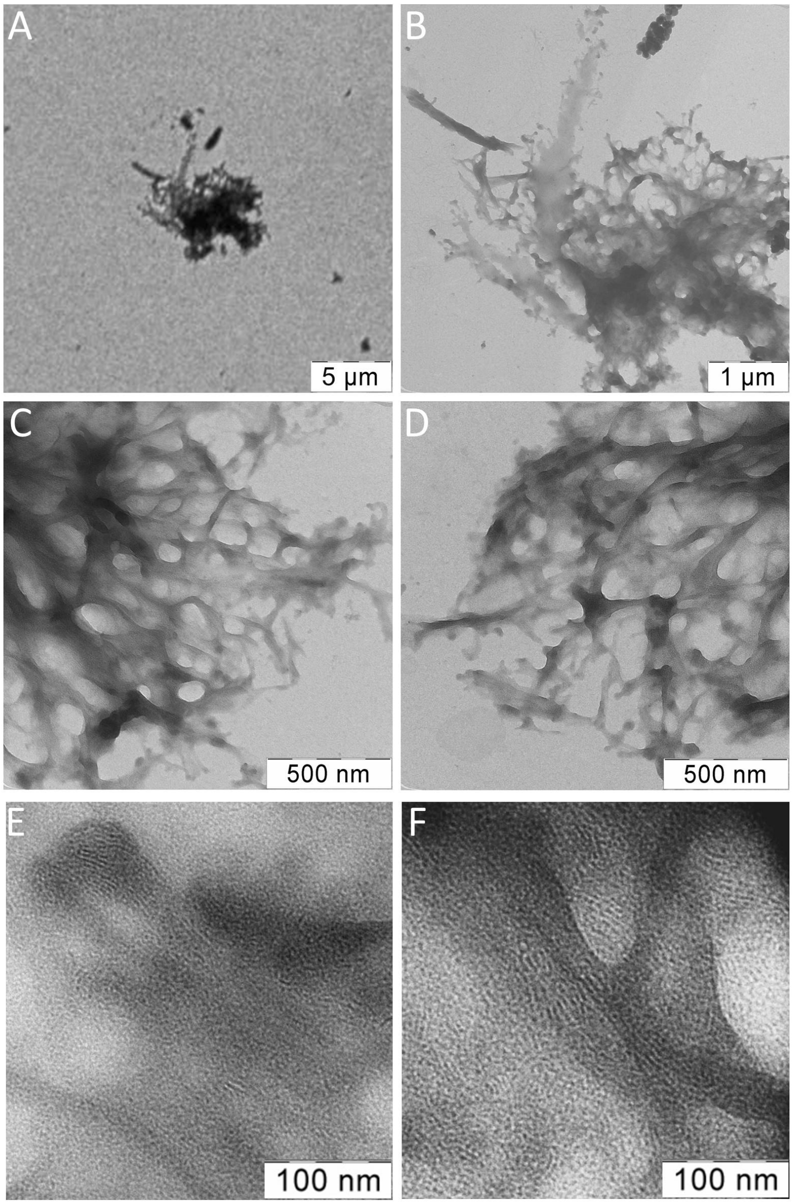

Figure S2. TEM of structures formed in G pool from *Saccharomyces cerevisiae* cell walls of *wt* strain. General view of jellyfish-like associates (**A**–**D**). Fibrillar structure of their bodies (**E**,**F**).

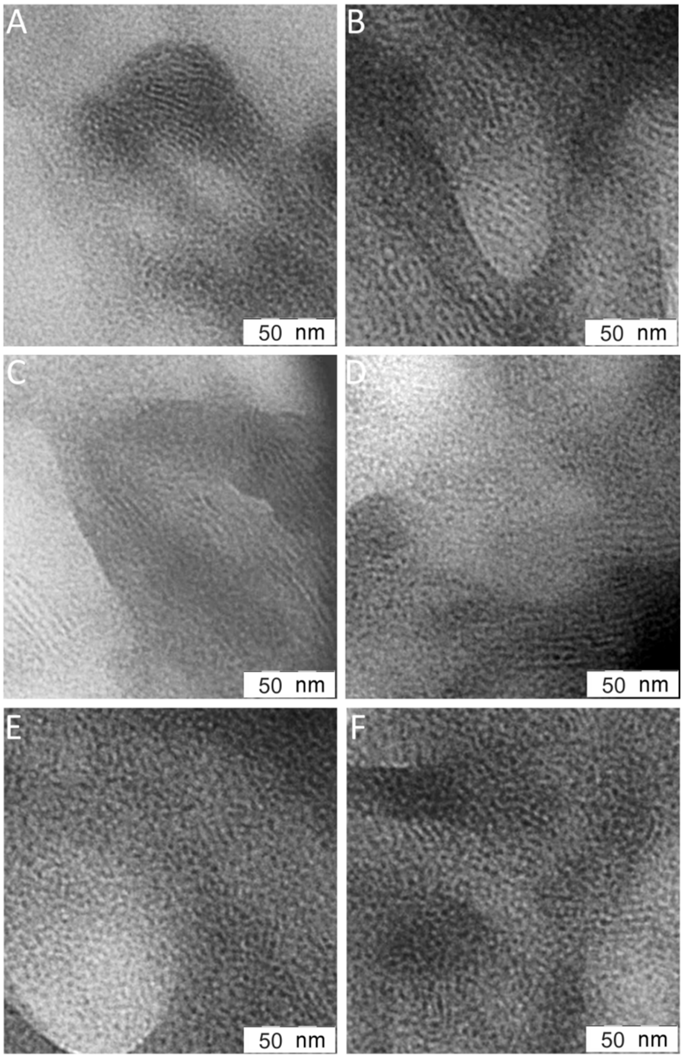

Figure S3. Fibrillar structure of jellyfish-like associates (**A**–**F**) of G pool from *Saccharomyces cerevisiae* cell walls of *wt* strain at high magnification.

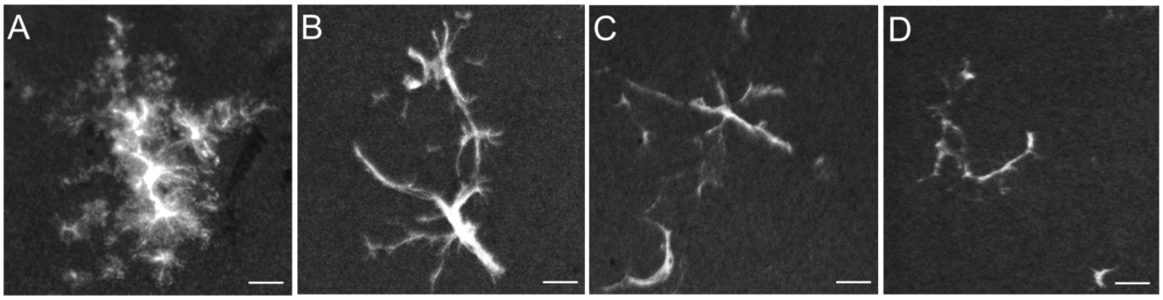

Figure S4. Immunofluorescence microscopy of structures (**A**–**D**) formed in G pool from *Saccharomyces cerevisiae* cell walls of *wt* strain stained with antibodies against Bgl2. Scale bars correspond to 5 μm.

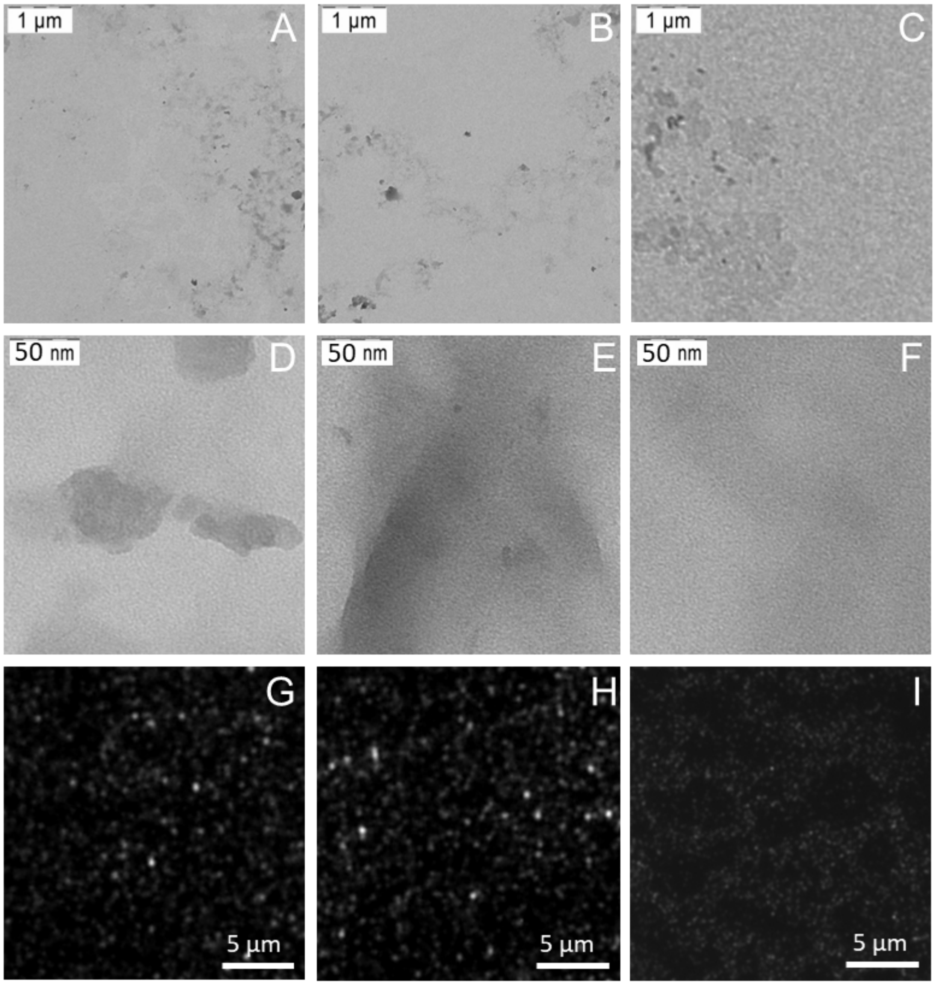

Figure S5. Microscopy of amorphous structures in T pool from *Saccharomyces cerevisiae* cell walls: TEM (**A**–**F**) and immunofluorescence confocal microscopy stained with antibodies against Bgl2 (**G**–**I**). Control samples from *bgl2Δ* strain (**C**,**F**,**I**).

Supplementary Data 3

Table S1. Comparison of amino acid residues in peptides lining the cavity near the active center of Bgl2 and the template protein 4wtp.1. The numbering of the amino acid residues (AA numbers) is indicated for the primary structure of Bgl2. Amino acid residues, highlighted in gray, constitute the cavity of the active center. Among them identical amino acid residues in the sequences of both proteins are highlighted in bold; underlined amino acid residues represent synonymous substitutions; remaining amino acid residues in the sequences of both proteins are non-synonymous substitutions.

AA numbers	30 - 36	61 - 67	90-93	123 - 130	166-170
Bgl2	**N**L**G**VKN**N**	KVY**A**ASD	**W**PTD	**SE**AL**YR**N**D**	VDSWN
4wpt.1	**N**Y**G**VNE**N**	RTF**A**LSV	**W**IDR	**SE**VL**YR**G**D**	ADVYY

AA numbers	190-195	232-235	274 - 295
Bgl2	**N**A**F**PY**W**	G**E**TG	**F**EAFD**E**DWKPNTSGTSDV**E**KHW
4wpt.1	**N**A**F**SY**W**	S**E**TG	**F**SAFD**E**PYR------GGV**E**AHF

Root-mean square deviation plot of Bgl2 trajectory is presented in Figure S6. Initial portion of 10 ns length was ignored for C40 to C68 sulfur-to-sulfur distance distribution and for estimation of fluctuations in the Bgl2 structure (Figures S7–S9).

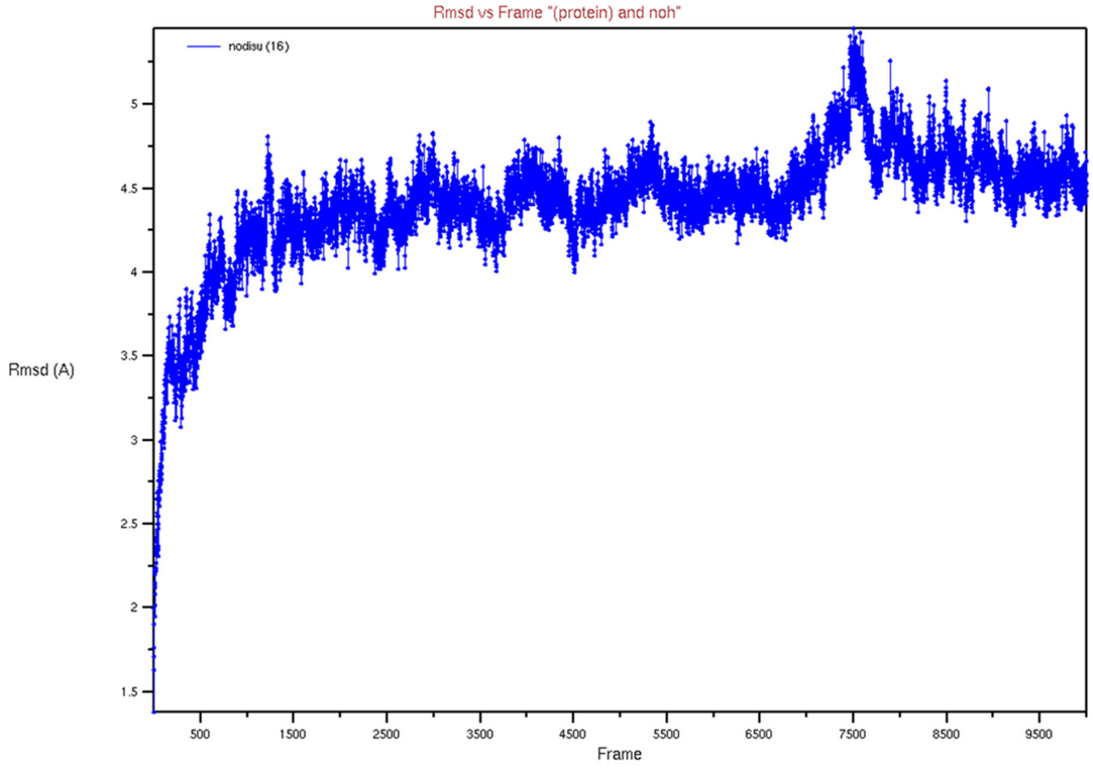

Figure S6. Root-mean square deviation (RMSD) plot of Bgl2 trajectory, with initial structure used as reference.

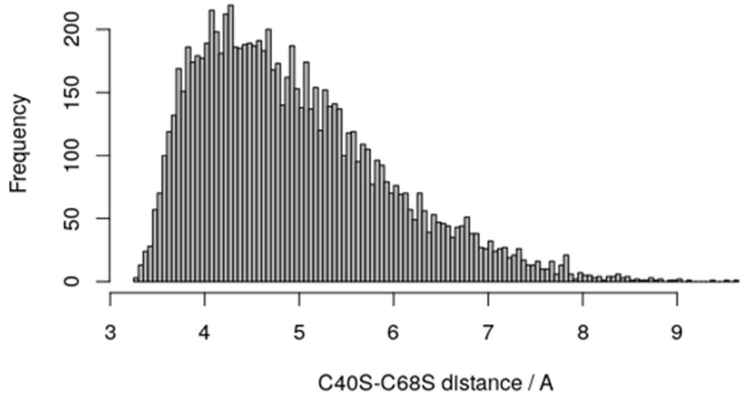

Figure S7. C40 to C68 sulfur-to-sulfur distance distribution. Max peaks are at 4–4.5 Å region, therefore, this linkage is presumably not critical for stability of the modeled protein fold.

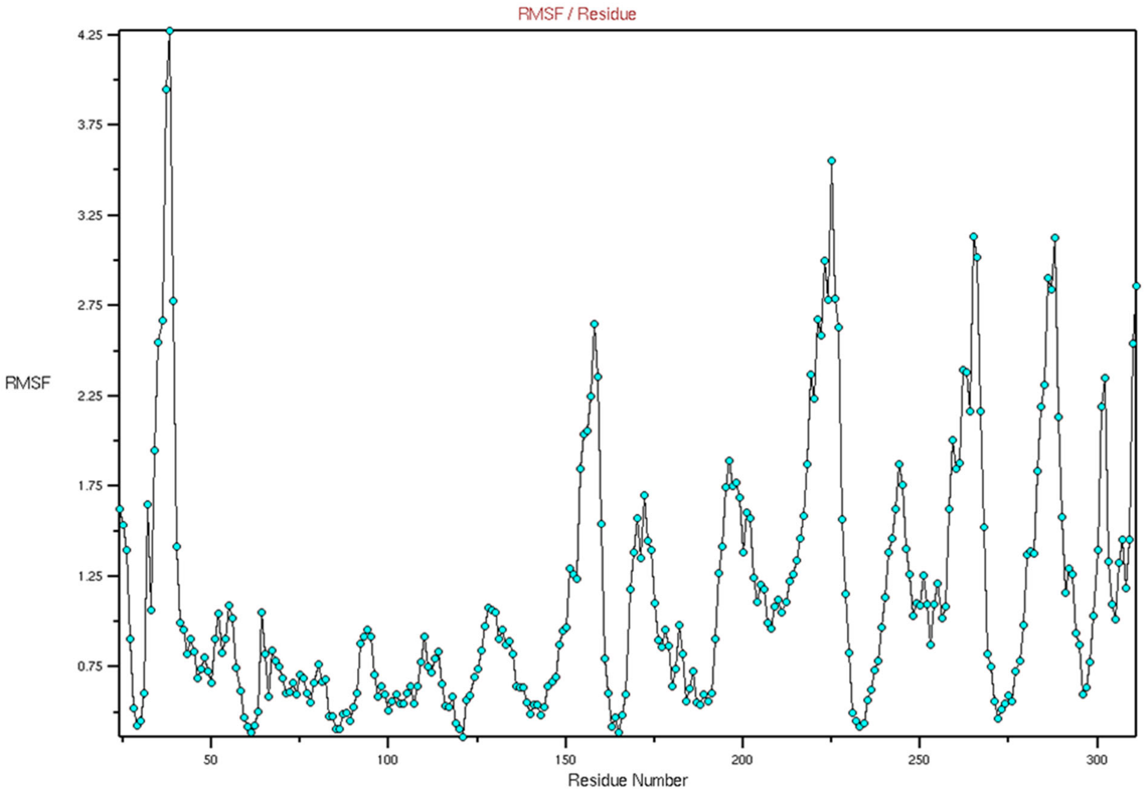

Figure S8. Per-residue root-mean square fluctuation (RMSF) plot of Bgl2 trajectory. Surface loops and some alpha helix ends are flexible regions. The beta-structured core is stable region.

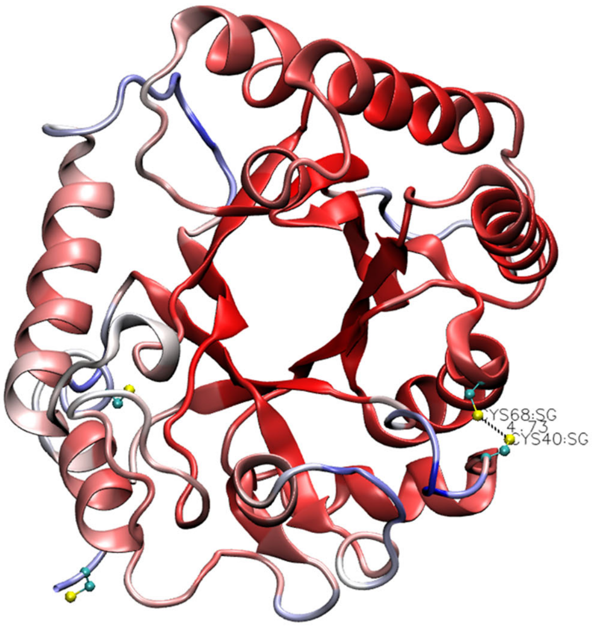

Figure S9. Bgl2 structure colored by root-mean square fluctuations. Red color designates less fluctuation, thus more stable structure.

Bgl2 backbone structure with glutathione was different from that without glutathione, and equilibration took significantly longer (Figures S10 and S11). Only last 40 ns of trajectory was shown for estimation of fluctuations in the Bgl2 structure (Figure S11).

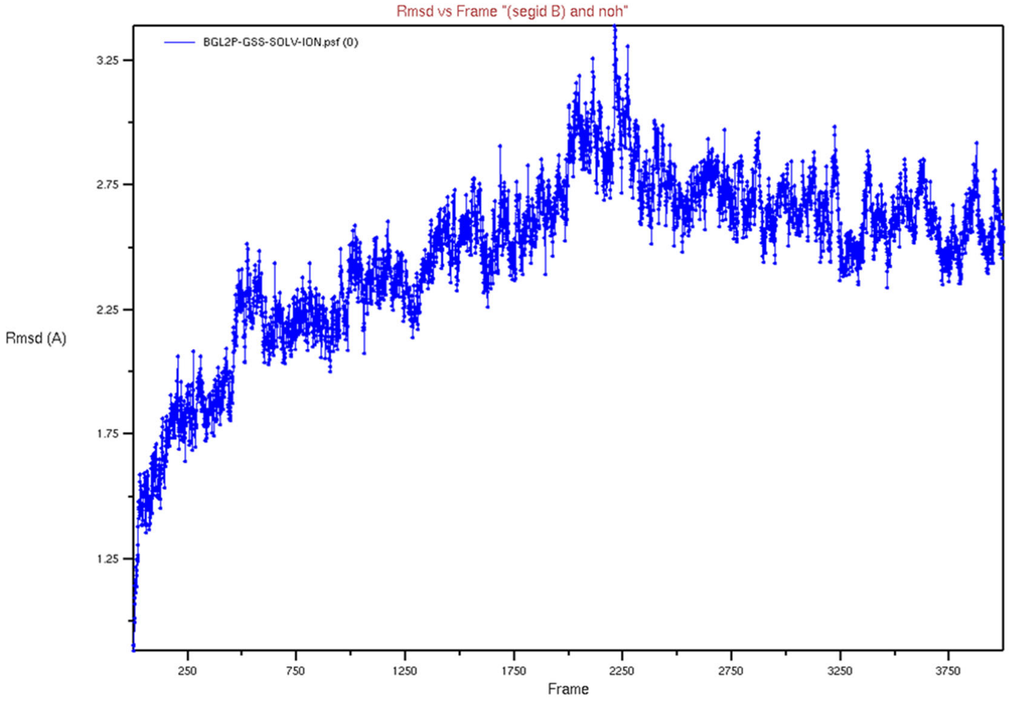

Figure S10. Root-mean square deviation (RMSD) plot of the trajectory of Bgl2 with glutathione molecule, with initial structure used as reference.

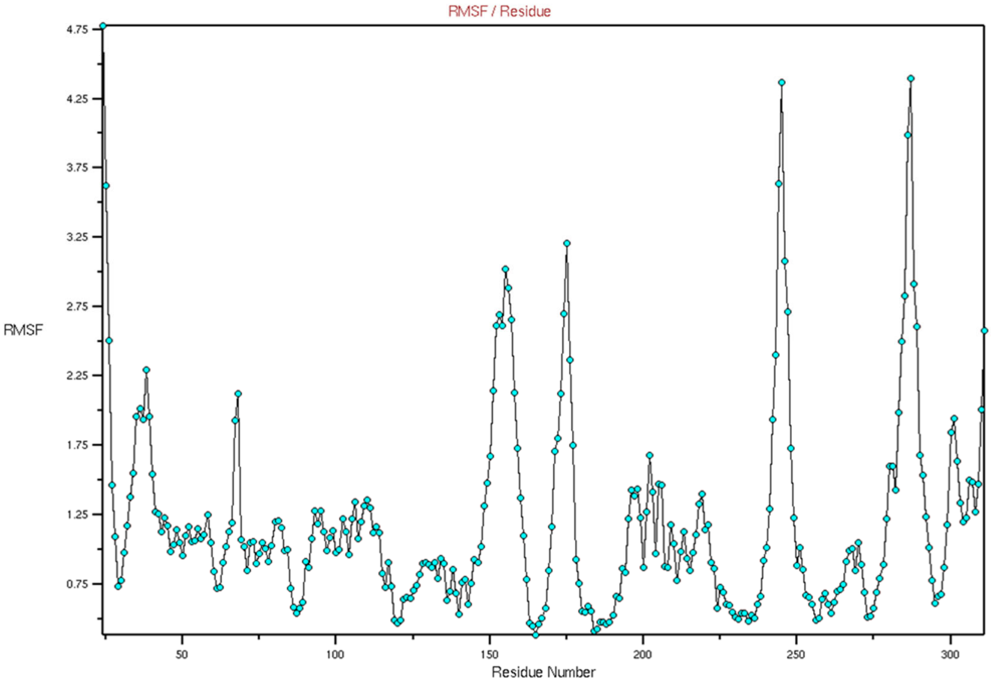

Figure S11. Per-residue root-mean square fluctuation plot of the trajectory of Bgl2 with glutathione molecule.

Most populated cluster of glutathione conformations in complex constructed by docking is presented in Figure S12. During steered molecular dynamics preferred glutathione conformation was found to stay underneath the G32-S42 loop (Figure S13). Distance from the glutathione positively charged amino group to the E233 was 15 Å on average. Despite the fact that glutathione can be located underneath the G32-S42 loop to approach position closer to the center of protein, the obtained conformations were not stable, and returned to the external glutathione conformation after the biasing potential was removed.

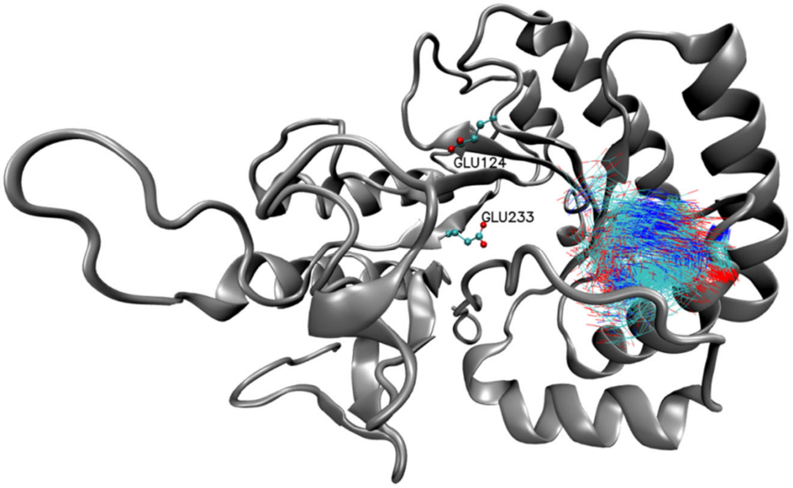

Figure S12. Most populated cluster of glutathione conformations in complex constructed by docking.

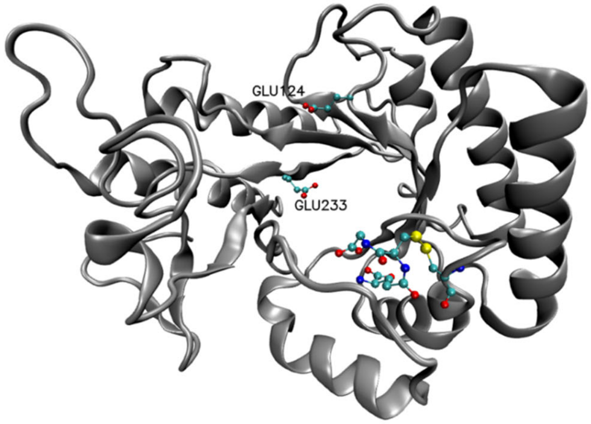

Figure S13. Representative structure of the most populated cluster of glutathione conformations in complex constructed by docking.

In the equilibrated structure of Bgl2 C68 was found to be significantly exposed to water, which allowed to locate glutathione externally. Corresponding model was built and equilibrated using the same procedures. Protein backbone was found to be stable (Figures S14 and S15). Glutathione was completely exposed to water (Figure S16). Distance from the glutathione positively charged amino group to the E233, positioned in close proximity to the center of Bgl2 molecule, was above 20 Å.

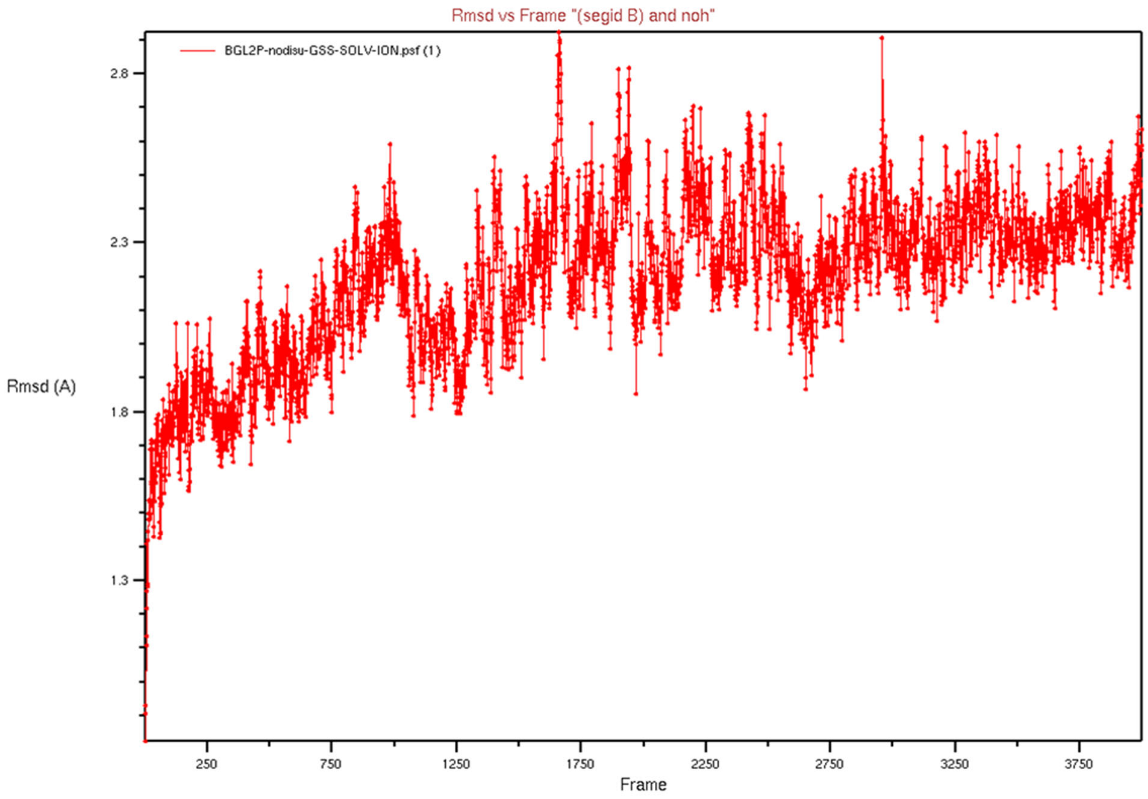

Figure S14. Root-mean square deviation (RMSD) plot of the trajectory of Bgl2 with glutathione molecule externally attached.

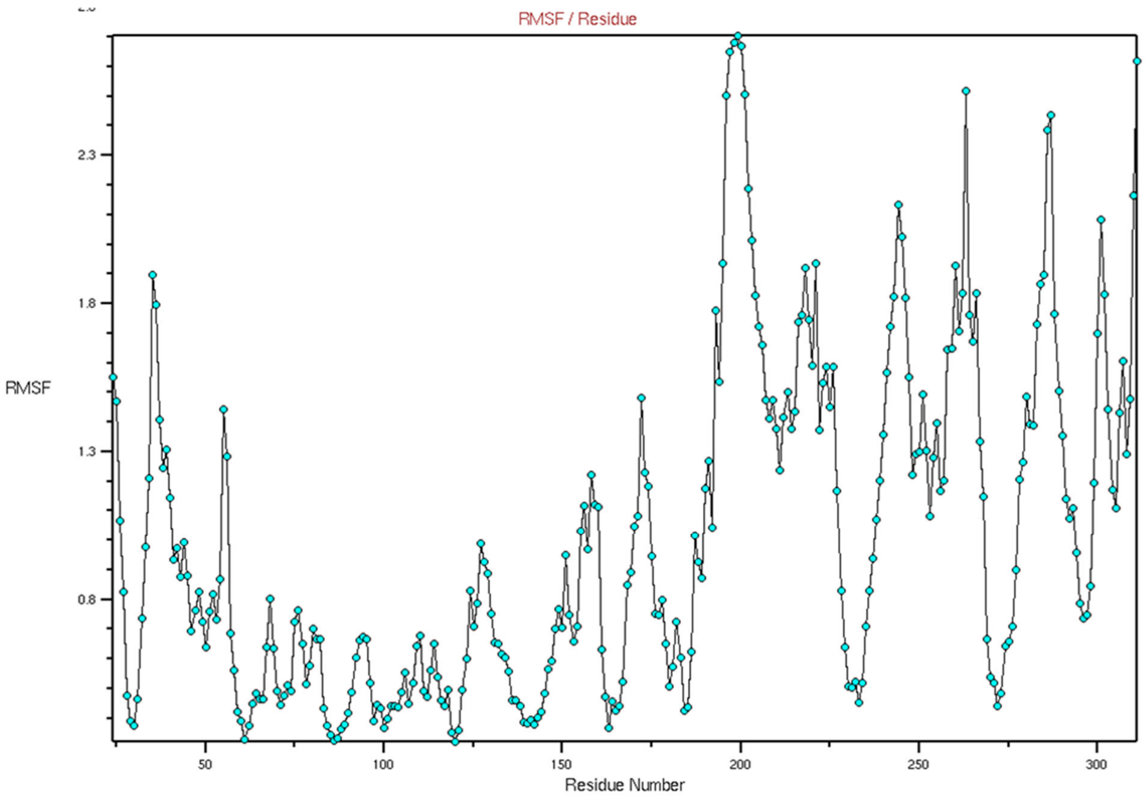

Figure S15. Per-residue root-mean square fluctuation plot of the trajectory of Bgl2 with glutathione molecule externally attached.

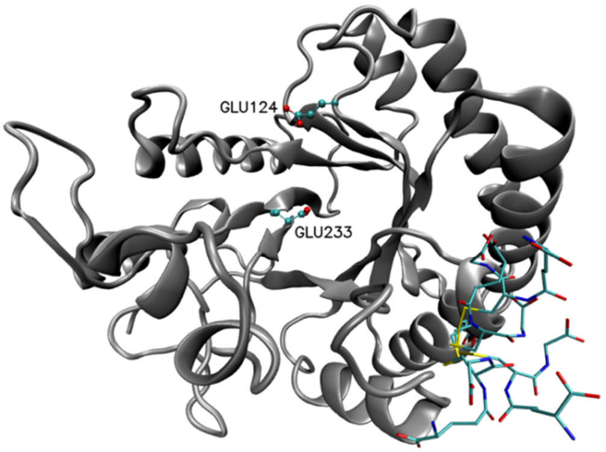

Figure S16. Conformations of glutathione attached externally to equilibrated model of Bgl2.

In course of trajectory, structure of Bgl2 phosphorylated at single T84 residue and corresponding to G pool, remained quite stable (Figures S17–S21). No significant changes of integral macromolecule parameters, such as solvent accessible surface area (Figure S19, black line) and radius of gyration (Figure S20, black line) were observed. On the contrary, fully phosphorylated model has undergone noticeable structural changes, and exhibited significant local structure flexibility (Figures S22 and S23), as well as growth of solvent accessible surface area (Figure S19, red line) and radius of gyration (Figure S20, red line) in course of dynamics. Taking into consideration that protein molecule itself carries pretty high negative charge (-20), addition of 10 negatively charged phosphate groups can easily destabilize the proper folding of the protein (Figure S24), and render it defunct, it is unlikely that protein really exists in fully phosphorylated state in T pool, and instead, what is present in T pool is a mixture of partially (or even singly) phosphorylated molecules, albeit at different OH-bearing amino acid residues.

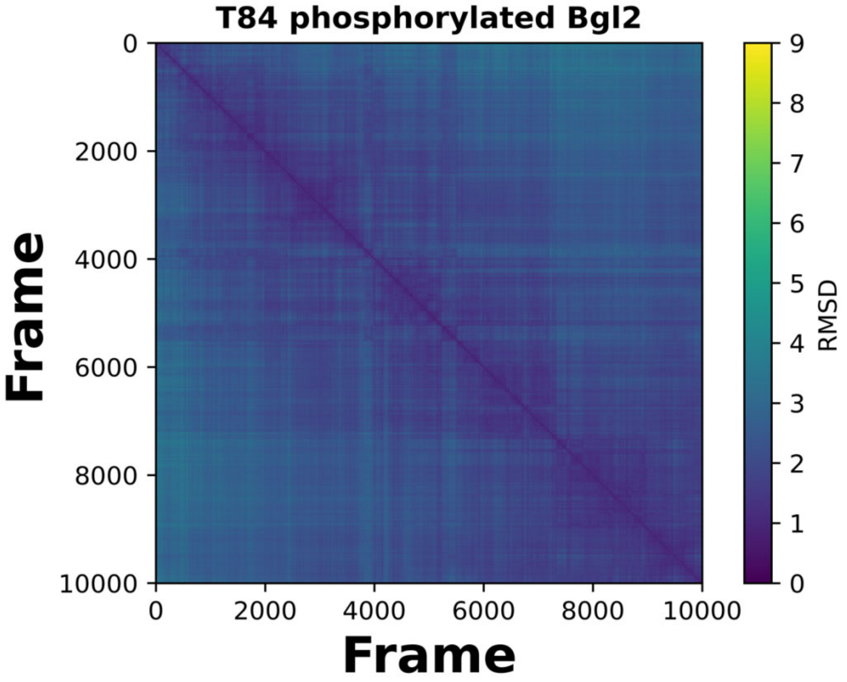

Figure S17. Heat map of root-mean square deviation (RMSD) plot of the trajectory of T84 phosphorylated Bgl2.

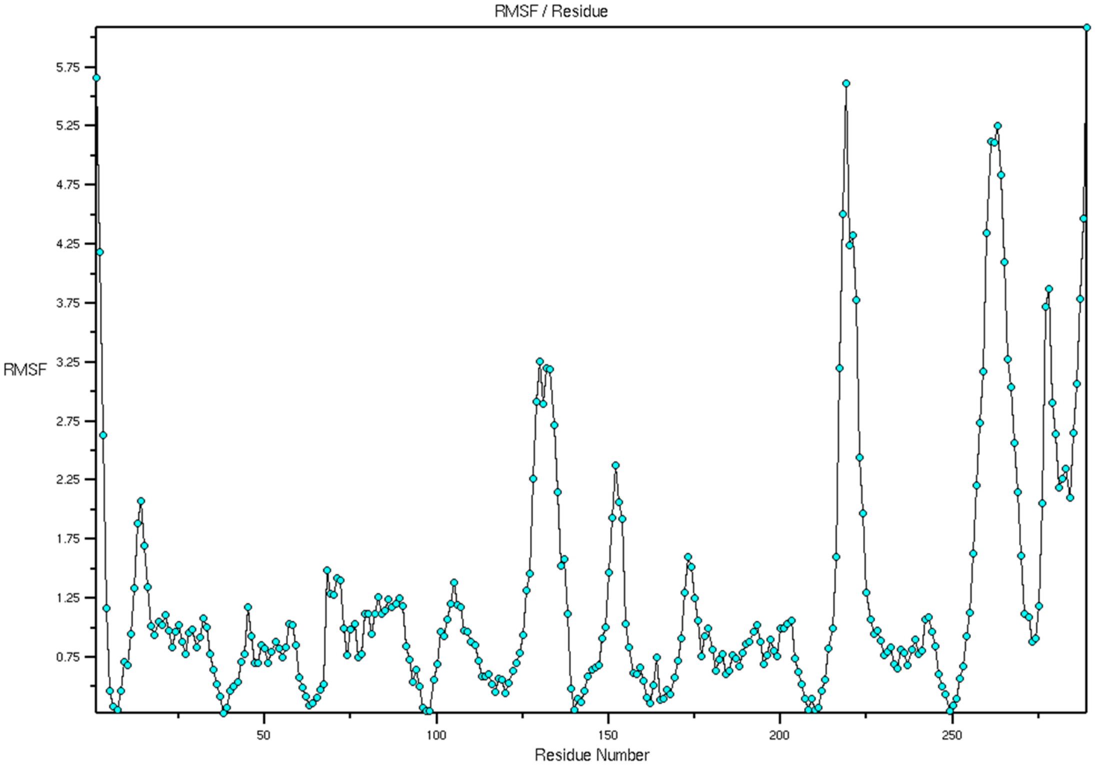

Figure S18. Per-residue root-mean square fluctuation (RMSF) plot of T84 phosphorylated Bgl2 trajectory.

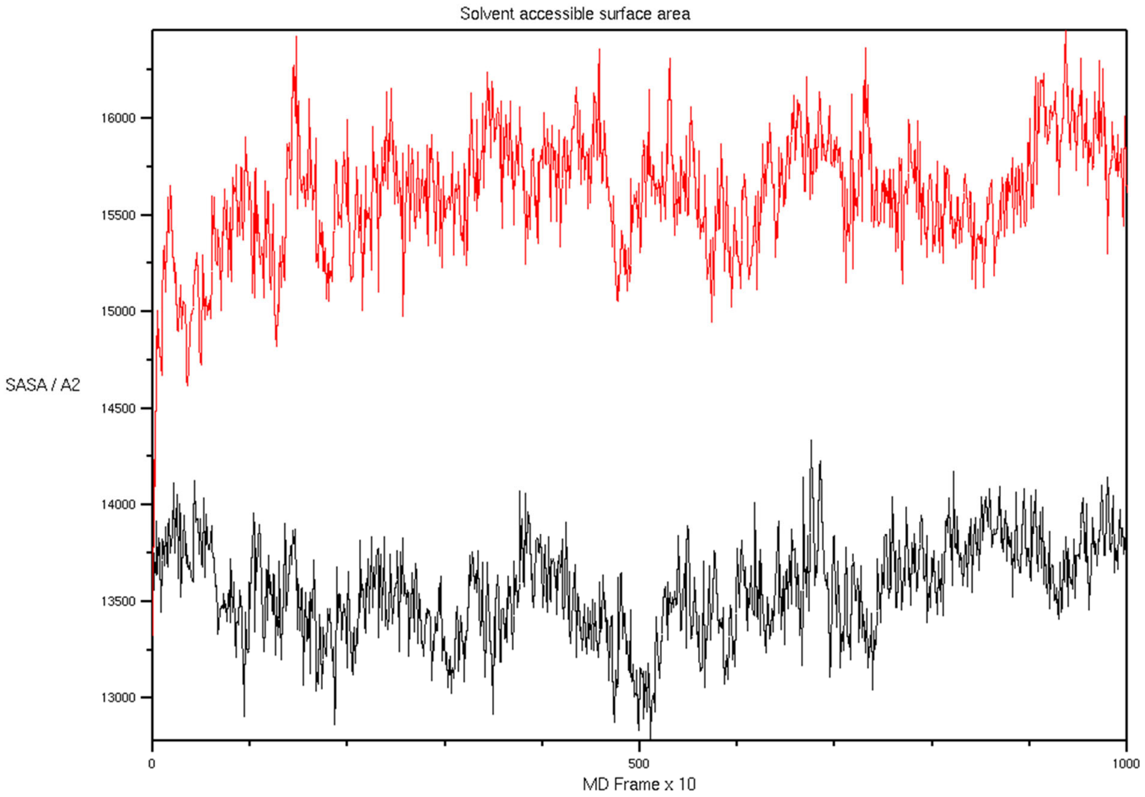

Figure S19. Solvent accessible surface area (SASA) of single phosphorylated at T84 (black line) and fully phosphorylated (red line) Bgl2 molecules. Full scale at X axis is 100 ns.

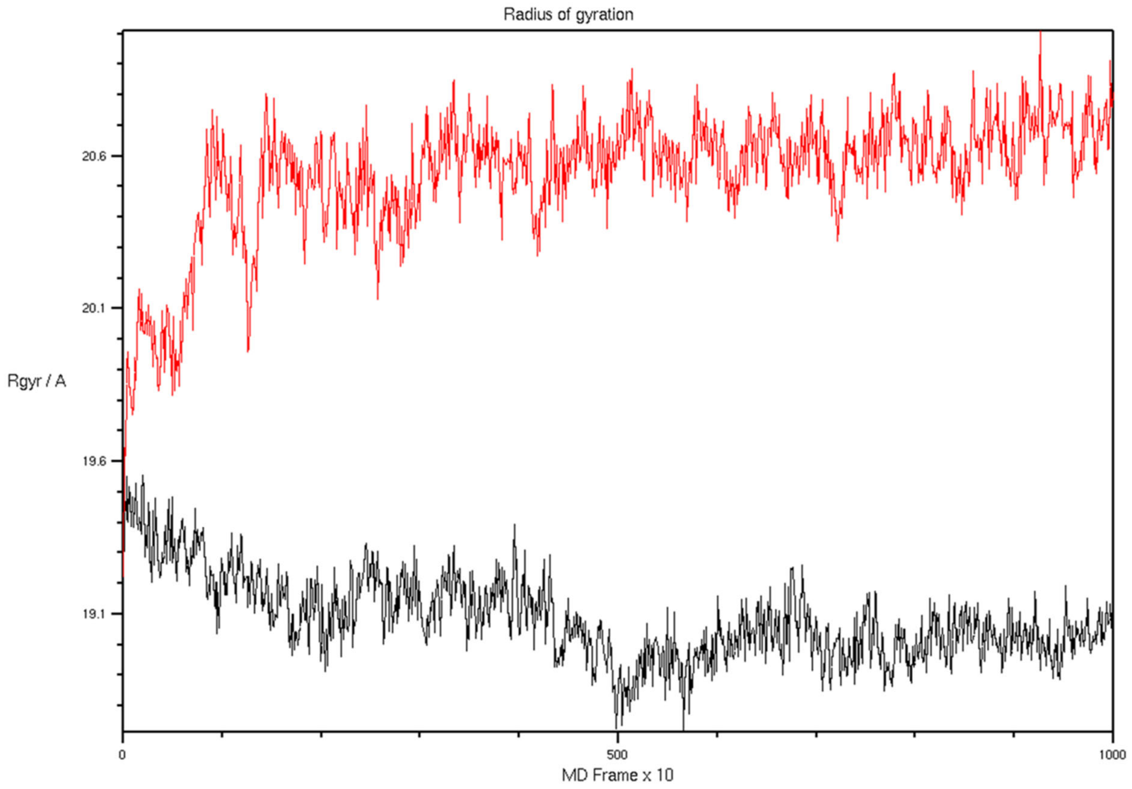

Figure S20. Solvent accessible surface area of single phosphorylated at T84 (black line) and fully phosphorylated (red line) Bgl2 molecules. Full scale at X axis is 100 ns.

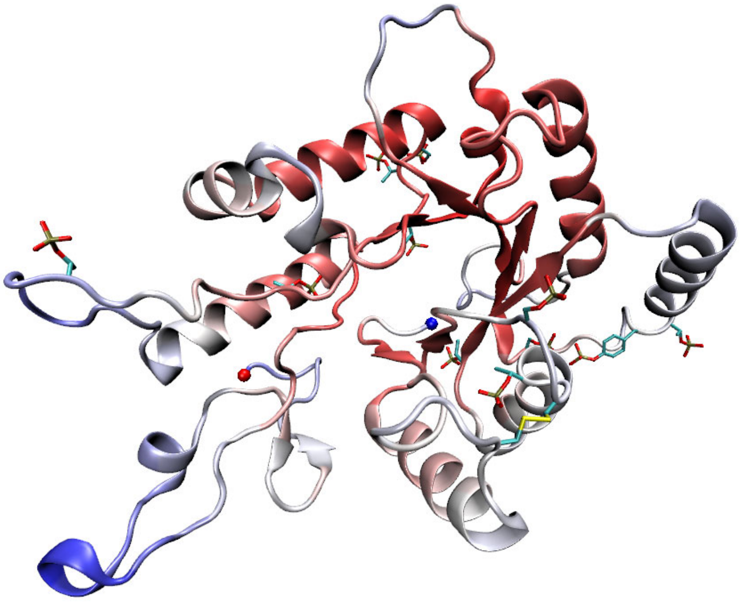

Figure S21. Model of fully phosphorylated Bgl2 trajectory after 100 ns, colored by root-mean square fluctuations. Red color designates less fluctuation, thus more stable structure. Phosphorylated residues are shown with sticks, red and blue balls indicate C- and N-terminus, correspondingly. It is clear that beta-barrel structure, intact in T84 phosphorylated protein, here is broken near C-terminal region.

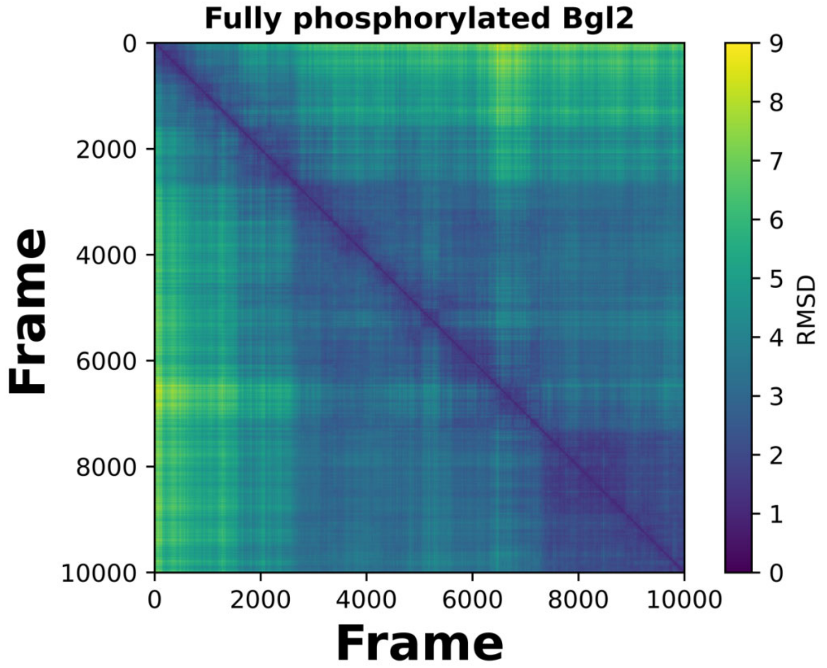

Figure S22. Heat map of root-mean square deviation (RMSD) plot of the trajectory of fully phosphorylated Bgl2.

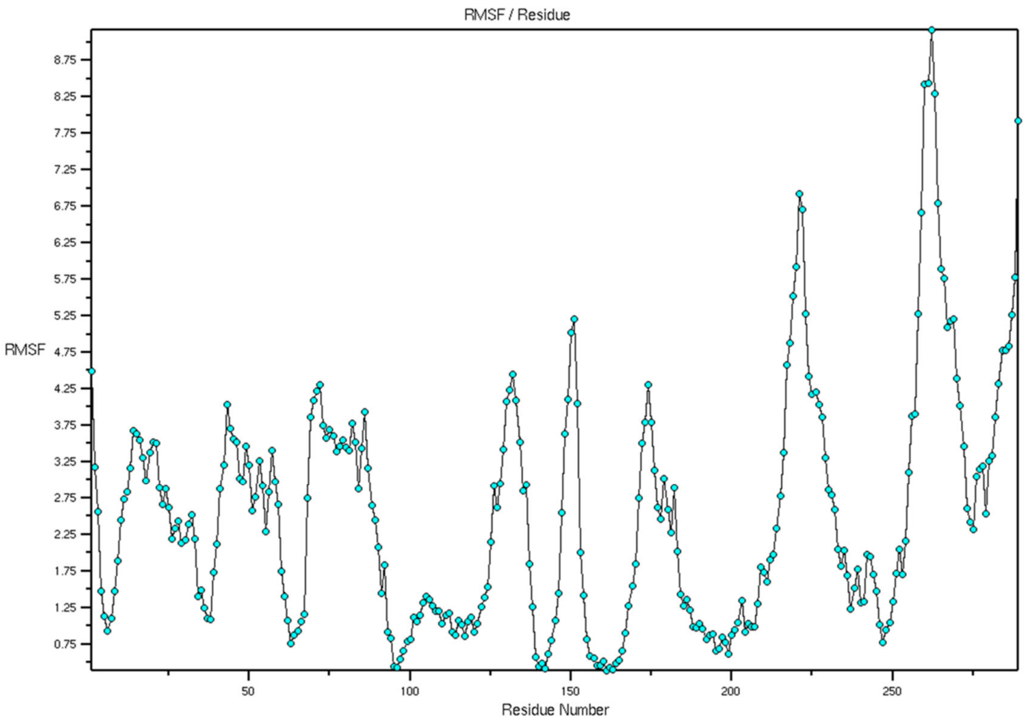

Figure S23. Per-residue root-mean square fluctuation (RMSF) plot of fully phosphorylated Bgl2 trajectory. Significantly higher RMSF values observed indicate both protein structure change and lower overall stability, especially near C-tail region, compared to unphosphorylated or T84-phosphorylated protein.

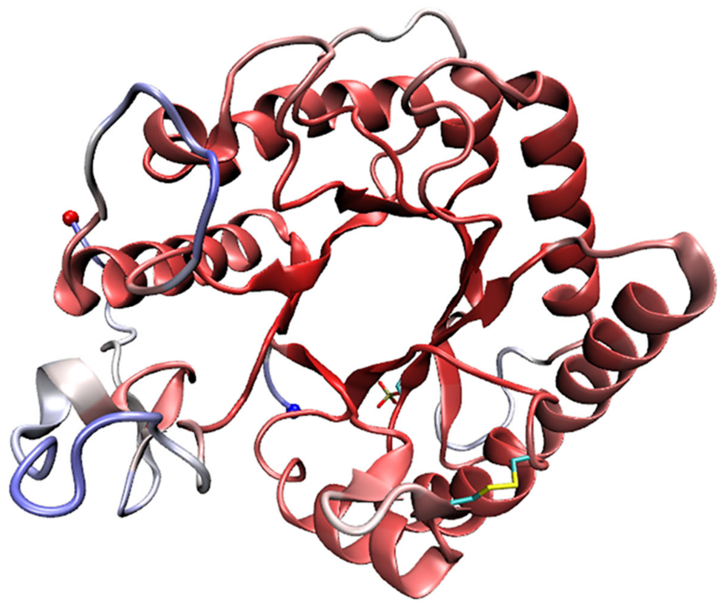

Figure S24. Model of T84 phosphorylated Bgl2 trajectory after 100 ns, colored by root-mean square fluctuations. Red color designates less fluctuation, thus more stable structure. Beta-barrel core is intact.

Supplementary Data 4

Investigation of Bgl2 co-localization with CW was carried out with the fluorescent confocal scanning microscope Carl Zeiss Axiovert 200M LSM 510 META (Zeiss, Germany). We failed to detect the presence and localization of Bgl2 in the CW not treated with glucanase (Figure S25), but we detected it after treatment of CW with glucanase (Figure S26). Figure S26 (panels C and F) demonstrates the co-localization of the fluorescent spots with the CW. The fluorescent images obtained with the fluorescent confocal scanning microscope Carl Zeiss Axiovert 200M LSM 510 META (Zeiss, Germany) are not so clear, but Figure S26 gives us the possibility to overlay fluorescent and phase contrast images of yeast CW. Thus the co-localization of the fluorescence and contours of CW is clearly visible. We also added an image in Figure S27 obtained with fluorescent confocal scanning microscope Leica TCS SP2 AOBS (Leica, Germany). Despite low magnification this image demonstrates a good co-localization of CW and fluorescent spots even in the absence of phase contrast, as such objects are bright and numerous.

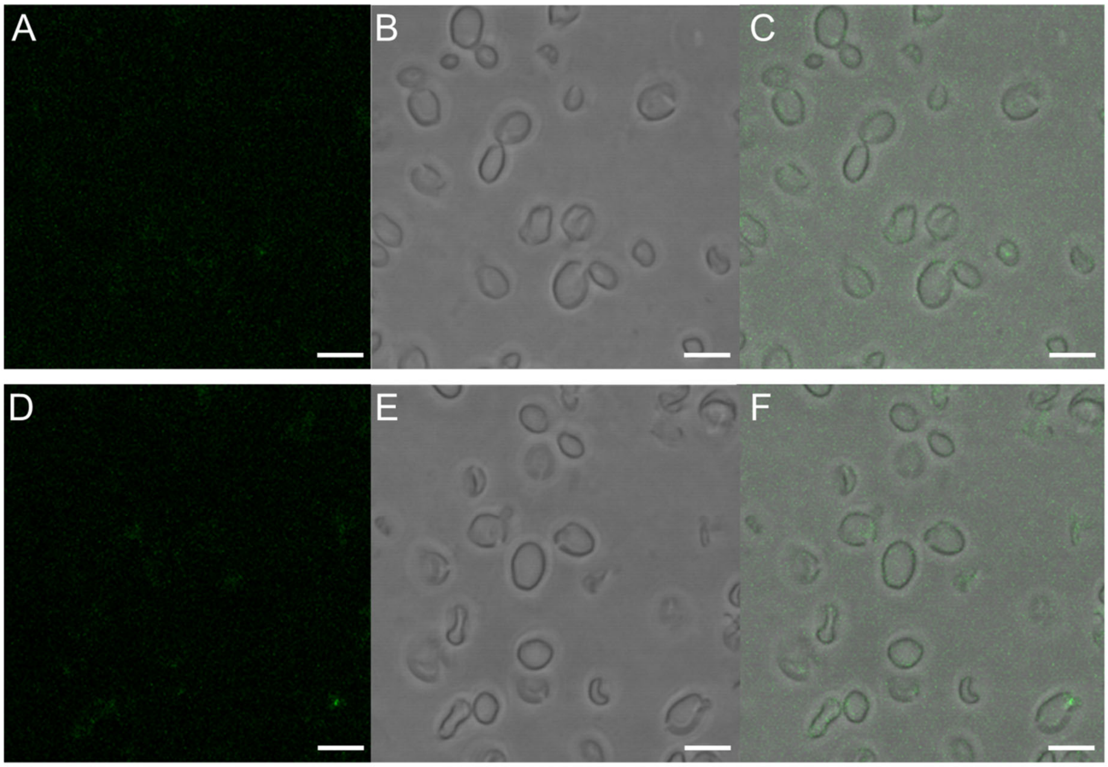

Figure S25. Immunofluorescence microscopy of untreated with glucanase *Saccharomyces cerevisiae* cell walls stained with antibodies against Bgl2. Scale bars correspond to 5 μm. (**A**) and (**D**) – fluorescent images, (**B**) and (**E**) – phase contrast images, (**C**) and (**F**) – overlay of the corresponding fluorescent and phase contrast images. Images were obtained with the fluorescent confocal scanning microscope Carl Zeiss Axiovert 200M LSM 510 META (Zeiss, Germany).

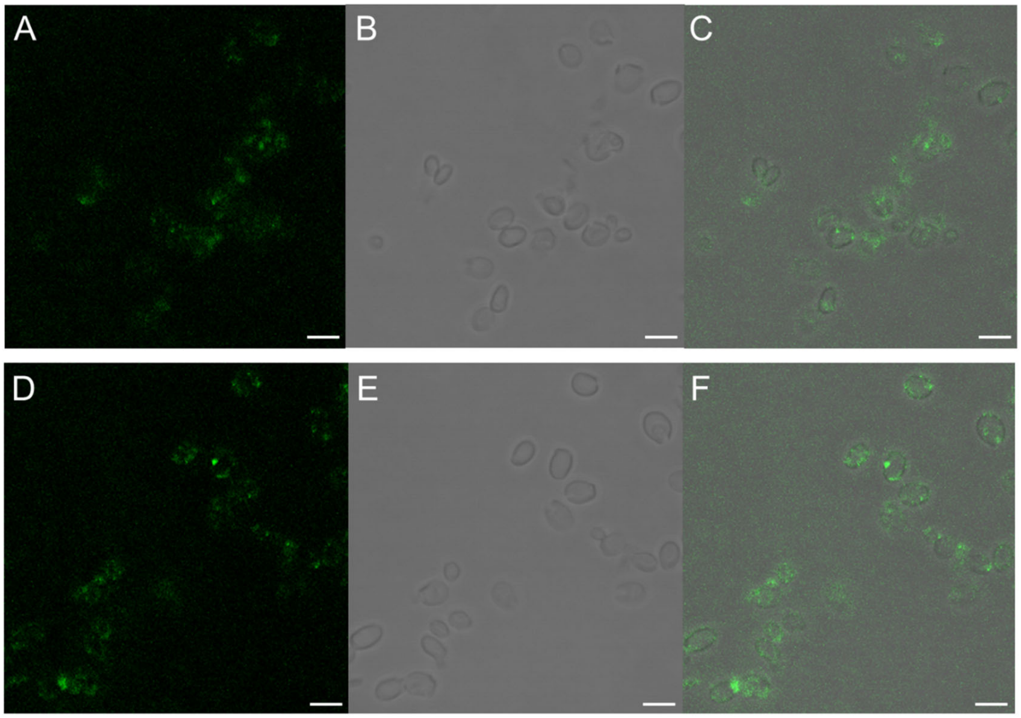

Figure S26. Immunofluorescence microscopy of glucanase-treated *Saccharomyces cerevisiae* cell walls stained with antibodies against Bgl2. Scale bars correspond to 5 μm. (**A**) and (**D**) – fluorescent images, (**B**) and (**E**) – phase contrast images, (**C**) and (**F**) – overlay of the corresponding fluorescent and phase contrast images. Images were obtained with the fluorescent confocal scanning microscope Carl Zeiss Axiovert 200M LSM 510 META (Zeiss, Germany).

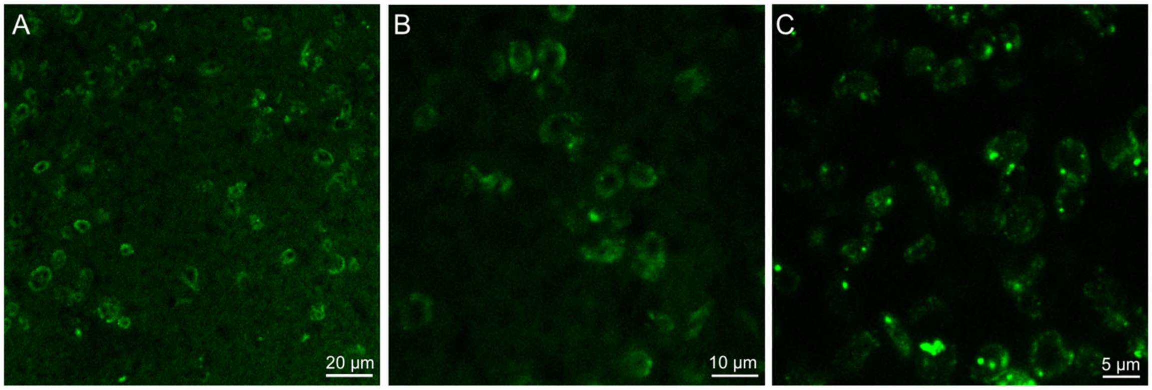

Figure S27. Immunofluorescence microscopy of *Saccharomyces cerevisiae* cell walls stained with antibodies against Bgl2 at different magnification. Scale bars correspond to 10 μm. Images were obtained with fluorescent confocal scanning microscope Leica TCS SP2 AOBS (Leica, Germany).

Supplementary Data 5

Another important ncGTGs Gas1, Gas3 and Gas5 were not detected in T and G pools. Nevertheless in this work and earlier [22] it was shown that these members of Gas family are present in CW as non-covalently bound proteins in L pool.

Gas1 incorporated into CW was previously visualized into the chitin ring and septum [57] while in the lateral CW Gas1 was not detected. Nothing is known about the distribution of Gas3 and Gas5 in the CW.

We visualized Gas proteins in the CW preparation stained with antibodies against Gas1. Gas1 has 38% identity with Gas3 and 44% identity with Gas5. Therefore, the possibility of recognition of Gas3 and/or Gas5 by antibodies to Gas1 cannot be ruled out.

Gas proteins in the CW preparation treated with EDC crosslinker and then boiled with 3% SDS (as described in subsection «CW protein crosslinking» of Material and methods) were visualized in the lateral CW (Figure S28 A-F). Optical sections of the CW preparation showed that Gas proteins can be localized mosaically (Figure S28 B, C, E, F). Gas proteins could not be detected without preliminary EDC crosslinking after boiling with 3% SDS (Figure S28 G-I).

Data obtained confirm that Gas proteins (most likely Gas1, but maybe also Gas3 and/or Gas5) as well as Bgl2 (Figure 5) can form microcompartments in the CW.

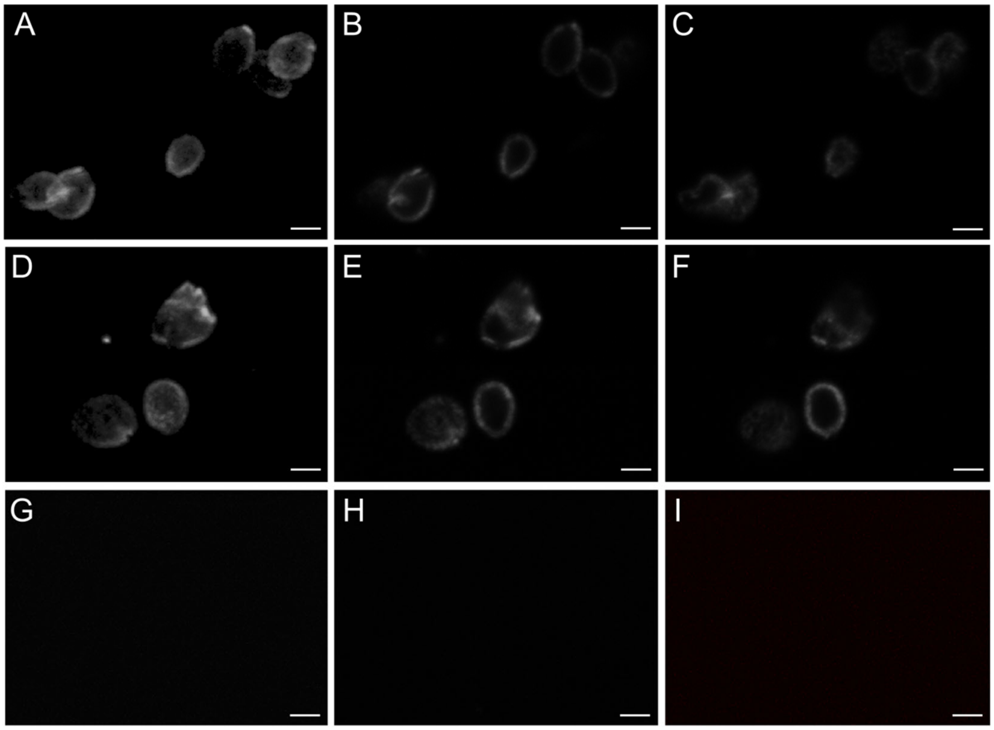

Figure S28. Immunofluorescence confocal microscopy of *Saccharomyces cerevisiae* cell walls with (**A**–**F**) and without EDC crosslinking (**G**–**I**). Staining with antibodies against Gas1. Scale bars correspond to 2 μm. (**B)** and (**C)** are different optical sections, corresponding to 3D projection (**A**). € and (**F**) are different optical sections, corresponding to 3D projection (**D)**. (**H**) and (**I**) are different optical sections, corresponding to 3D projection (**G**).
